# Engineered cell therapies for autoimmune diseases: evaluating CAR-T and CAR-M progress and prospects

**DOI:** 10.3389/fimmu.2025.1658867

**Published:** 2025-11-20

**Authors:** Mingxuan Zheng, Jiale Chen, Hong Yang

**Affiliations:** School of Medical and Life Sciences, Chengdu University of Traditional Chinese Medicine, Chengdu, Sichuan, China

**Keywords:** CAR-T, CAR-M, autoimmune diseases, macrophages, RA, SLE

## Abstract

Autoimmune diseases (AID) comprise a diverse group of disorders driven by aberrant B-cell and T-cell reactivity against self-tissues. In recent years, cell-based therapies utilizing engineered T cells have emerged as a promising therapeutic strategy for AIDs. Notably, chimeric antigen receptor (CAR)-T cells have demonstrated the ability to selectively target and eliminate autoreactive immune populations, including pathogenic B cells and antibody-producing plasma cells. Beyond T-cell modulation, macrophages (MΦs) exhibit remarkable plasticity, differentiating into pro-inflammatory (M1) or anti-inflammatory (M2) phenotypes in response to microenvironmental cues. Advances in genetic engineering have enabled the development of CAR-MΦs (CAR-M), which hold potential for adoptive immunotherapy in certain diseases. However, CAR-M therapy remains experimental and requires further clinical validation. This review systematically evaluates the therapeutic potential of CAR-T and CAR-M in AIDs, comparing their respective advantages and limitations to provide a comprehensive foundation for future translational applications.

## 1 Introduction

Autoimmune disease (AID) management is rapidly evolving from broad immunosuppression toward selective immune reprogramming. Chimeric antigen receptor (CAR)-based approaches employ genetically engineered immune cells, such as T cells and macrophages (MΦs), expressing CARs to recognize and eradicate target cells presenting specific antigens (1). First-generation CAR concepts emerged in the 1980s (2). Clinically, CAR-T cells targeting CD19 and other B-cell antigens have achieved notable success in relapsed/refractory B-cell malignancies, spurring exploration of CAR-T therapy in AIDs, including systemic lupus erythematosus (SLE), where CD19-targeted CAR-T cells have reduced anti-dsDNA antibodies with no notable adverse events reported (3). Unlike conventional T cells, CAR-T cells recognize antigens via an extracellular CAR binding domain, providing MHC-independent recognition and signaling through tandem intracellular modules (e.g., CD3ζ with CD28) for activation and costimulation in a single receptor (4). CAR-T cells are classified into autologous (autoCAR-T), derived from the patient, and allogeneic (alloCAR-T), derived from a donor; autologous CAR-T offers high compatibility with reduced graft versus-host disease (GvHD) risk but faces manufacturing delays and costs (5).

CAR-engineered MΦs (CAR-M) may rebalance M1/M2 polarization and foster anti-inflammatory responses. Mechanistic studies identify signaling domains such as Megf10, FcRγ, and PI3K p85 that promote phagocytosis, with tandem signaling enhancing engulfment (6). Preclinical work in humanized models laid the groundwork for AID applications (7), and Elite CAR-M showed efficacy in rheumatoid arthritis models, supporting safety signals (8). However, CAR-M in AID remains largely preclinical, with translational pathways still needed.

The field must balance between efficacy and safety. CAR-M in AID is early and largely preclinical, whereas CAR-T therapies are more mature and have been explored across several AIDs including rheumatoid arthritis (RA), SLE and Sjögren’s syndrome (SS) (3, 8, 9). Safety concerns include cytokine release syndrome (CRS), immune effector cell–associated neurotoxicity syndrome (ICANS), and GvHD; IL-6 is a central CRS mediator and target of tocilizumab. Long-term safety, immune reconstitution, and durable responses require more data. Before broad clinical adoption, scalability, accessibility, and health-system integration must be addressed (10, 11). In sum, this review highlights the potential of CAR-T and CAR-M in AID and advocates continued exploration to translate preclinical gains into safe, scalable therapies.

## 2 CAR-T cell engineering

### 2.1 CAR-T cell manufacturing and structural design

**Figure 1:**
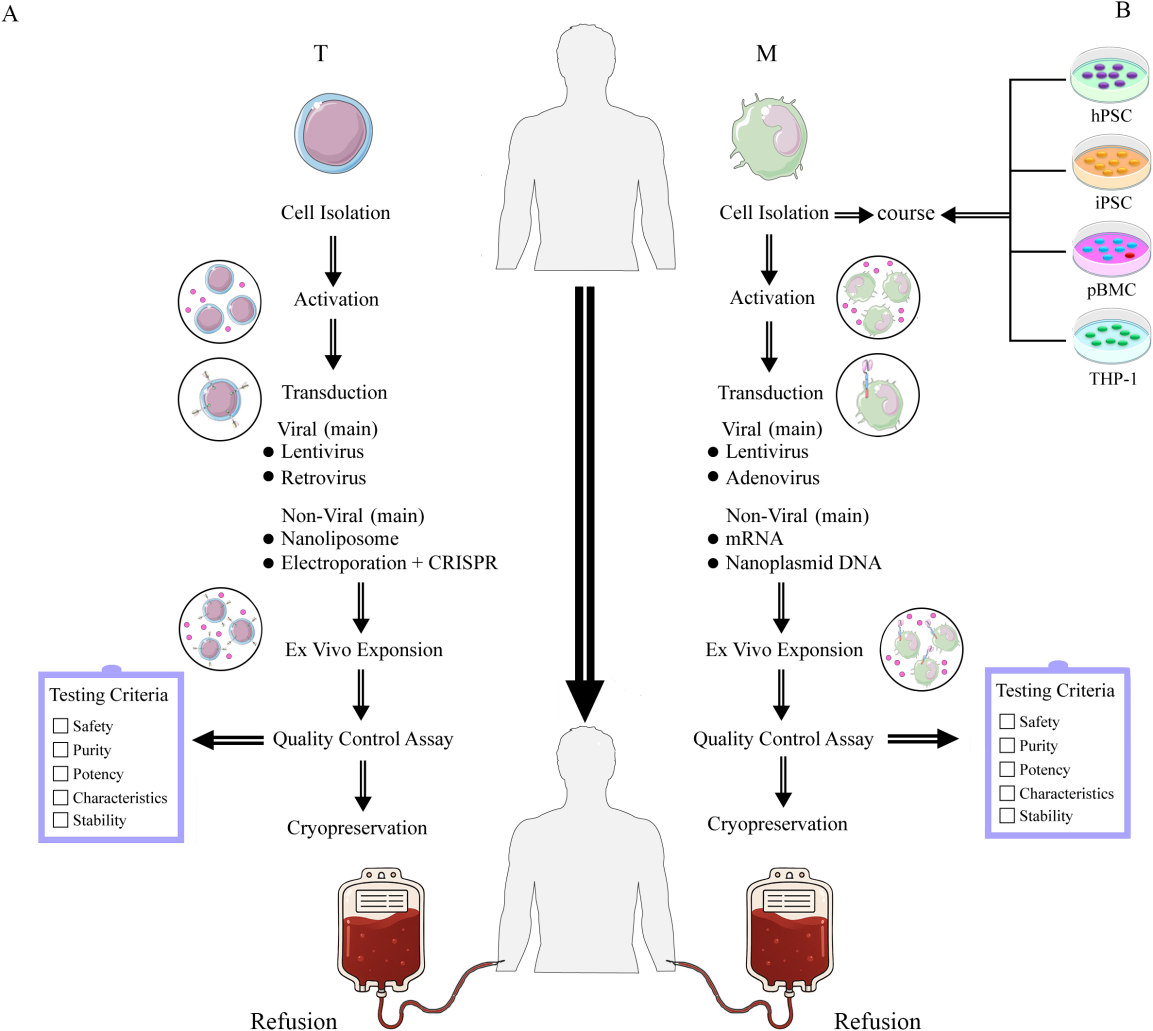
The manufacture of CAR-T **(A)** and CAR-M **(B)** cell therapies involves distinct isolation and transduction strategies for different cell types. **(A)** Manufacture of CAR-T. The left panel **(A)** illustrates the T cell processing workflow, encompassing cell isolation, activation, transduction, and *in vitro* expansion, followed by quality control assessment before reinfusion into the patient. **(B)** Manufacture of CAR-M. The right panel **(B)** demonstrates application workflows using human pluripotent stem cells (hPSCs), induced pluripotent stem cells (iPSCs), peripheral blood mononuclear cells (PBMCs), and THP-1 cells as starting cell lines. Macrophages (M) undergo cell isolation, activation processing, transduction, and *in vitro* expansion, followed by quality control assessment. Upon passing quality control, they are reinfused into the patient.

*Ex vivo* CAR-T production comprises five stages with rigorous quality controls to ensure consistency and safety (12). Stage 1 isolates and activates T cells from either healthy donors (alloCAR-T) or patients (autoCAR-T) via leukapheresis (13), followed by enrichment and standardized activation with beads coated with anti-CD3 and anti-CD28 antibodies. Stage 2 constructs and transduces the CAR, comprising (1) an antigen-recognition domain (scFv), (2) an extracellular hinge, (3) a transmembrane domain, and (4) an intracellular signaling domain; current FDA-approved products employ lentiviral or retroviral transduction to achieve stable CAR expression (14). Stage 3 expands CAR-T–expressing T cells in cytokines (e.g., IL-2, IL-7, IL-15) and fetal bovine serum (FBS) to reach therapeutic doses while depleting non-T cells, typically over 1–2 weeks (15). Stage 4 evaluates safety, purity, potency, functionality, and stability per established clinical criteria (16, 17). Lastly, the fifth stage involves cryopreservation and storage of the CAR-T cell products, which are preserved in a liquid state at -80°C in a liquid nitrogen freezer; recent advances include DMSO together with hydroxyethyl starch (HES) for optimized viability (18), **Figure 1A**.

### 2.2 Generations of CAR-T cells

**Figure 2:**
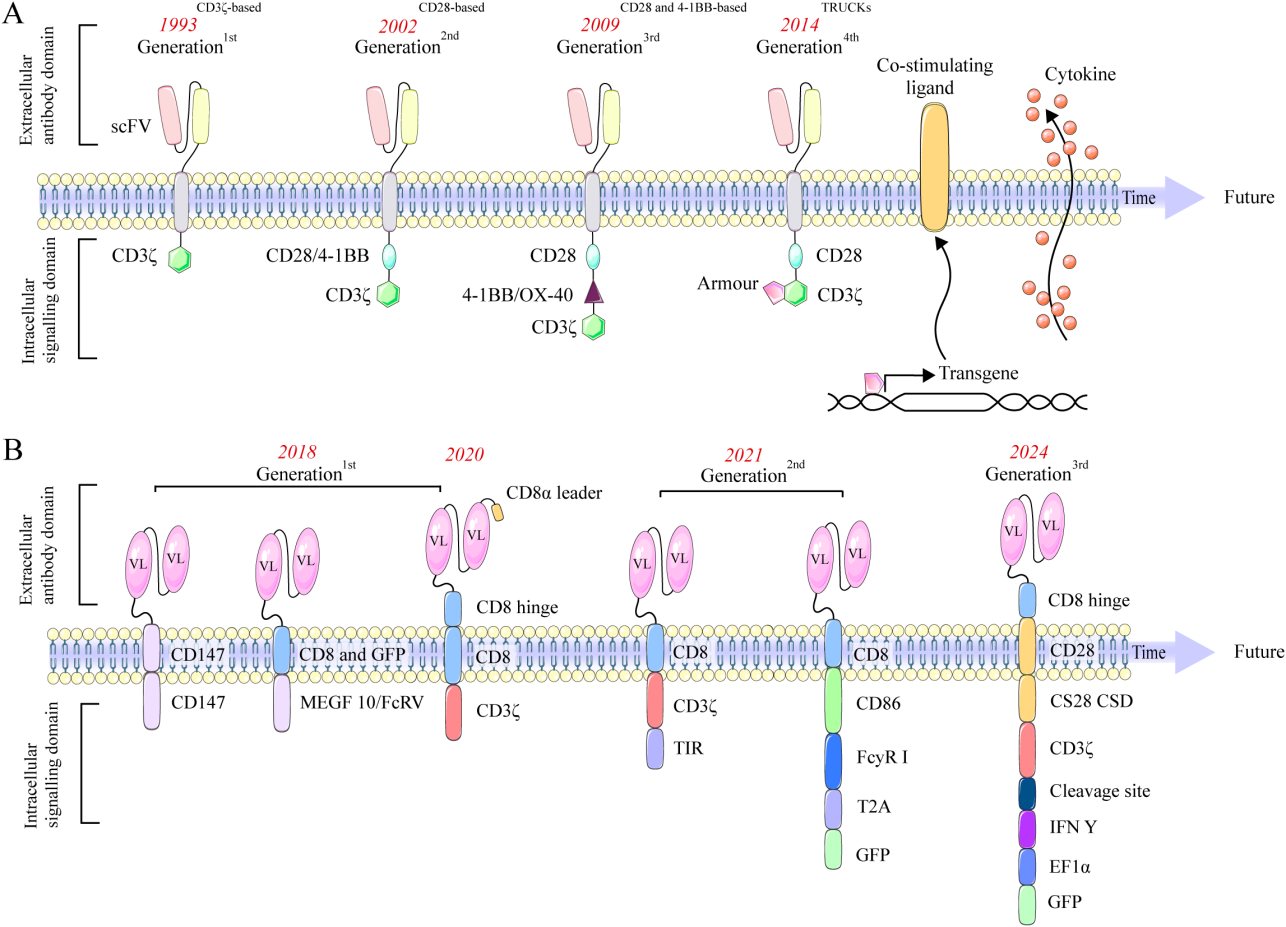
Evolution of CAR-T **(A)** and CAR-M **(B)** designs across generations, showing changes in structural domains and signaling elements. **(A)** Evolution of CAR-T cells. The first generation features a CD3ζ domain. The second adds a single co-stimulatory domain, such as CD28 or 4-1BB, enhancing T cell activation. The third generation builds upon this by incorporating two co-stimulatory domains, further improving signaling strength and efficacy. The fourth, or TRUCK, integrates IL-12 and an NFAT domain to boost cytokine production. The fifth incorporates cytokine receptor domains for better expansion and sustained activity. **(B)** Evolution of CAR-M. First-generation CAR-Ms targeted antigens and polarized MΦs to an M1 phenotype. Second-generation CAR-Ms used Ad5f35 vectors to convert M2 to M1, enhancing T cell activation. CAR-iMacs, derived from iPSCs, demonstrated antigen-dependent phagocytosis, while dual-signal CARs further boosted M1 polarization. Third-generation CAR-Ms employed nanobiotechnology to reprogram M2 into M1, enhancing anti-tumor activity.

Five generations of CAR-T cells have been developed. The first-generation CAR-T prototype developed nearly four decades ago, incorporates a CD3ζ signaling domain in its intracellular region (19). The second generation, in addition to the CD3ζ signaling domain, incorporates a co-stimulatory domain, typically comprising the intracellular domains of CD28 or 4-1BB (CD137). The third generation features two co-stimulatory domains. The fourth generation is developed by incorporating interleukin (IL)-12 into the second-generation construct framework and is designated as T cell redirected for general cytokine-mediated killing (TRUCK) (20). Additionally, fourth-generation CAR-T cells are engineered with an intracellular domain containing a nuclear factor of activated T cells (NFAT) domain. This design enhances the secretion of cytokines, including IL-12, IL-15, and granulocyte-macrophage colony-stimulating factor (GM-CSF), which mediates T cell activation (21). Recent advancements in fifth-generation CAR-T technology enable enhanced *in vitro* expansion and prolonged cytotoxic activity *in vivo* by incorporating intracellular domains with cytokine receptors. Specifically, these CAR-T cells feature the truncated cytoplasmic domain of the IL-2 receptor beta chain (IL-2Rβ), which activates the JAK-STAT signaling pathway, promoting cell proliferation and preventing terminal differentiation *in vitro* (22, 23), **Figure 2A**.

## 3 CAR-M engineering

### 3.1 CAR-M manufacturing and structural design

MΦ are traditionally framed as M1 (classically activated) and M2 (alternatively activated), though polarization exists along a functional continuum, reflecting dynamic states rather than discrete categories (21). Mantovani et al. further subdivided M2 into M2a (wound healing), M2b (regulatory), M2c (acquired inactivated), and M2d (tumor-associated MΦs) (24). M1 cells express CD68, CD80, CD86, MHCII, and iNOS, whereas M2 secrete IL-6, IL-10, and TGF-β, shaping a Th2-type milieu.

CAR-M therapy manufacturing follows core CAR-T steps but differs in cell origin, separation methods, and transduction strategies (25). MΦ sources include PBMC-derived MΦs (most accessible) but with lower gene-editing efficiency and clinical feasibility challenges (26). Alternative origins encompass induced pluripotent stem cells (iPSCs), human embryonic stem cells (hESCs), and the THP-1 monocytic line (26). CAR-M share the canonical CAR architecture: an extracellular antigen-recognition domain, a hinge, a transmembrane domain, and an intracellular signaling module. CD3ζ is a common signaling component for both CAR-T and CAR-M; additional CAR-M–relevant modules include CD147, FcRγ, and Megf10 (6, 7). Gene transfer increasingly relies on lentiviral or adenoviral vectors (e.g., Ad35; often combined with Ad5F35 to enhance lymphocyte infection and overcome MΦ resistance), supporting sustained M1 polarization and improved efficacy (27). Post-transduction, CAR-M are expanded *in vitro* (commonly with FBS and GM-CSF) and CAR expression is verified (e.g., GFP by flow cytometry) (28), as illustrated in **Figure 1B**.

### 3.2 Generations of CAR-M

The first-generation CAR-M utilize intracellular signaling domains such as Megf10 or CD147. Morrissey et al. pioneered enhanced phagocytic capabilities using Megf10 (6), while Zhang et al. developed CAR-HER2 constructs that employ CD147 to activate matrix metalloproteinases, enabling extracellular matrix degradation to overcome physical barriers (29). In addition, Klichinsky et al. reported notable clinical activity in a Phase I trial of anti-HER2 CAR-M for recurrent/metastatic HER2-overexpressing solid tumors (NCT04660929) (7). The second generation of CAR-M incorporates co-stimulatory domains. Lei’s tandem CD3ζ-TIR dual signaling design yields induced MΦs (iMAC) with enhanced phagocytosis, antigen-dependent M1 polarization, and NF-κB–dependent resistance to M2 polarization (30). Zhang et al. reprogrammed donor PBMCs into iPSCs, added FcγR co-stimulation and CAR via lentivirus to produce CAR-iMACs with >50× expansion, >30 days persistence, and CAR expression up to 85% (31). Third-generation CAR-M is defined by its molecular design, which incorporates cytokine-based co-stimulatory domains and utilizes non-viral *in vivo* reprogramming strategies, representing an emerging paradigm in cellular engineering (32). Zhou et al. used lipid nanoparticles to deliver Trop2-CAR plasmid DNA (LNP/CAR-Trop2) for *in situ* CAR-M generation after intravenous administration; Trop2-CAR-Ms selectively target Trop2-overexpressing tumors and promote NK and CD8 T cell proliferation, with peak IL-6 levels ~one-fifth of those seen with conventional CAR-T and no serious CRS events (33). This *in vivo* reprogramming strategy presents a promising approach to circumvent the complexities of *ex vivo* CAR manufacturing and mitigate potential systemic toxicities. Duan et al. designed a CAR-M targeting VEGFR2, showing upregulated CD86, MHCII, and TNF-α *in vitro* and substantially inhibited tumor progression in 4T1-bearing mice without notable toxicity (34). Together, these studies highlight two key innovations in third-generation CAR-M: cytokine signaling modules and non-viral *in vivo* delivery, as shown in **Figure 2B**.

## 4 CAR-T cell therapy: an emerging therapeutic strategy for autoimmune diseases with perspectives on CAR-M

**Figure 3:**
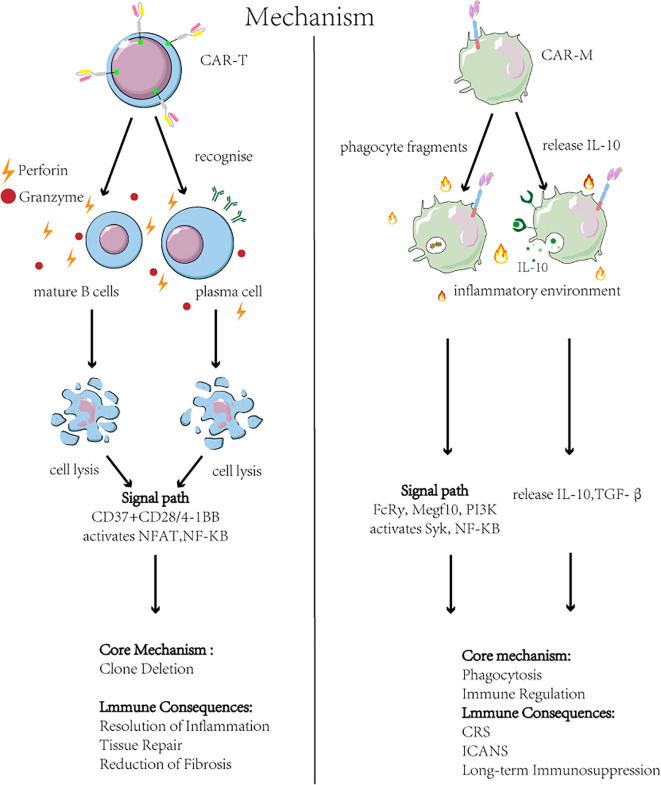
Mechanisms and outcomes of CAR-T and CAR-M therapies in autoimmune disease. Left: CAR-T cell therapy targets B-cell antigens (e.g., CD19/BCMA), triggering cytotoxic elimination of pathogenic B cells and plasma cells via NFAT/NF-κB activation and perforin/granzyme release. This achieves B cell depletion and reduced autoantibodies but risks CRS and neurotoxicity ICANS. Right: CAR-Macrophage (CAR-M) therapy engages antigens on apoptotic debris or profibrotic cells, inducing phagocytosis (via Syk) and anti-inflammatory polarization (via PI3K/NF-κB). CAR-M clear debris and secrete IL-10/TGF-β, promoting inflammation resolution and tissue repair. Key challenges include short persistence and hepatic sequestration.

Although CAR-T and CAR-M therapies are designed with similar chimeric antigen receptor constructs, their core immunological mechanisms of action in AID are fundamentally distinct (35), as illustrated in **Figure 3**. The efficacy of CAR-T therapy primarily stems from the clonal deletion of pathogenic immune cells. Clinical studies confirm its capacity to precisely deplete CD19^+^ B cells, resulting in a marked reduction or seroconversion of autoantibodies (e.g., anti-dsDNA) (3, 36). Critically, an immune reset is observed post-treatment, characterized by the reconstitution of a native B cell repertoire, which may underpin the establishment of long-term immune tolerance (36). This is accompanied by a concomitant reduction in inflammatory cytokines, indicating a recalibration of the cytokine network (37, 38). In contrast, CAR-M therapy does not primarily mediate direct cytotoxicity. Instead, it leverages the innate functions of engineered macrophages to remodel the local tissue microenvironment. The following sections will elaborate on their specific applications across various autoimmune diseases within this mechanistic framework.

### 4.1 Autoimmune arthritis

RA is driven by T lymphocytes, with collagen, citrullinated collagen (cit C), and chondrocytes playing pivotal roles in disease pathogenesis (39). Collagen, a major extracellular matrix protein, acts as a natural ligand for LAIR-1, whose activation suppresses autoimmune responses (40). HLA-DR alleles confer genetic risk in RA development. Collagen type II (Col2a1), linked with HLA-DRB1/DRA1 expression, has been used to target and eliminate CII-specific CD4 T cells, reducing RA in murine models (41). Citrullination of collagen (cit C) is a fundamental RA mechanism; cit C is detected in RA synovium and is a primary target of autoantibodies (ACPA) in serum and synovial fluid (42, 43). As a competitive inhibitor, cit C binds LAIR-1, dampening LAIR-1–mediated inhibition and potentiating autoimmunity (40). Targeting or depleting cit C presents a novel therapeutic approach, including anti-FITC CAR-T cells directed against FITC-labeled citrullinated peptides to selectively target self-reactive B cells, though this remains preclinical (44). The chondrocyte–T cell interaction drives RA; Liu et al. created Col2a1-CAR-T cells (Col2a1-CAR with scFv-CD137-CD3ζ) via lentiviral infection, which secrete TNF-α and induce IL-6 in chondrocytes, highlighting the need for regulatory mechanisms to prevent excessive activation in cartilage-immune interactions (45).

Acute RA is associated with increased M1 activity. Therapeutic strategies aimed at reducing M1 and promoting M2 polarization show promise in alleviating disease severity (46–48). CAR-M has also made significant progress in RA. IL-10 serves as a crucial mediator in M2 polarization. Clustered regularly interspaced short palindromic repeats transcriptional activation (CRISPRa) exploits endogenous cellular mechanisms to effectively upregulate the expression of endogenous genes. Huang integrated the CRISPRa system with CAR-M-based adoptive immunotherapy to generate engineered, durable IL-10 receptor CAR-M (Elite M). Elite M enhances IL-10 secretion, thereby inducing a pre-activated state in M2 through an autocrine mechanism. Additionally, it produces paracrine signals that inhibit the release of pro-inflammatory mediators from M1, thereby promoting chondrocyte differentiation. In a mouse model of RA, CRISPRa-engineered Elite MΦs significantly reduced immune cell infiltration, synovial hyperplasia, and joint destruction. Furthermore, the absence of significant *in vivo* toxicity observed in the RA mouse model supports the safety profile of this approach (8), **Table 1**.

**Table 1:** Summary of CAR-based therapeutic targets, cell types, and experimental models applied to autoimmune diseases.

CAR	Target antigen	Targeted cell	Disease/pathology	Experiment type	Citation	Trial numbers
CAR-T	BCMA	B cell	SLE	AEM	(50)	
CAR-T	BCMA	B cell	MG	CT	(64)	NCT04146051
CAR-T	BCMA	B cell	MG	CT	(65)	NCT04561557
CAR-T	BCMA	B cell	IMNM	CT	(66)	NO
CAR-T	BCMA and CD19	B cell	MG	CT	(62)	NO
CAR-T	CD19	B cell	SLE	*In Vitro*	(52)	
CAR-T	CD19	B cell	SLE	*In Vitro*	(38)	
CAR-T	CD19	B cell	SLE	CT	(75)	NO
CAR-T	CD19	B cell	refractorySLE	CT	(3)	NO
CAR-T	CD19	B cell	refractorySLE	CT	(51)	NCT05859997
CAR-T	CD19	B cell	SjD	CT	(9)	NO
CAR-T	CD19	B cell	MG	CT	(63)	NO
CAR-T	CD19	B cell	MS	AEM	(71)	
CAR-T	CD19	B cell	MS	CT	(129)	NCT05859997
CAR-T	CD19	B cell	Myositis associated With Antisynthetase Syndrome	CT	(67)	NO
CAR-T	CD19	B cell	ITP	AEM	(89)	
CAR-T	TSHR	B cell	GD	AEM	(80)	
CARR-T	TSHR	B cell	GD	*In Vitro*	(81)	
CAR-T	Citrullinated peptide epitopes	B cell	RA	*In Vitro*	(44)	
CAR-T	XCR1	DC	EAE	AEM	(72)	
CAR-T	pMHCII	CD4 T cell	EAE	AEM	(69)	
CAR-T	pMHCII	CD4 T cell	T1D	AEM	(76)	
CAR-T	DR1-CII	CD4 T cell	Autoimmune Arthritis	AEM	(41)	
CAR-T	CII	chondrocyte	RA	*In Vitro*	(45)	
CAR-Treg	MOG	CD8 T cell	EAE	AEM	(70)	
CAR-T	PD-1	CD8 T cell	PBC	AEM	(85)	
CAR-M	IL-10	Macrophage	RA	AEM	(8)	
CAR-M	uPAR	HSC	Fibrosis	AEM	(88)	
CAR-M	FAP	HSC	Fibrosis	AEM	(89)	
CAR-M	FAP	cardiac myocyte	Fibrosis	AEM	(87)	
CAR-eM	BAI1	NPC	–	*In Vitro*	(92)	

*In Vitro* refers to experiments conducted outside a living organism, specifically using human cells in this context.

CT, Clinical Trial; AEM, Animal Experimentation with Mice.

No: This experiment did not have an NCT.

### 4.2 Systemic lupus erythematosus

In SLE, B cell–targeted CAR-T strategies (CD19, BCMA) show early preclinical efficacy and favorable safety signals; preclinical data support anti-CD19 CAR-Ts with 4-1BB, and BCMA-expressing B cell targeting (APRIL-based CAR-T) prolongs survival in SLE models, underpinning ongoing clinical exploration (49, 50). The therapeutic potential of CAR-T therapy in SLE was first demonstrated in a landmark study by Mougiakakos et al., who reported the successful use of anti-CD19 CAR-T cells to treat a patient with refractory SLE and lupus nephritis, achieving serological remission including seroconversion of anti-dsDNA antibodies in the absence of significant adverse events, thereby establishing a foundational milestone for the field (3). This proof-of-concept has since been corroborated and extended in larger clinical investigations. A recent study involving individuals with severe, refractory SLE and multi-organ involvement demonstrated that all three patients treated with CD19-directed CAR-T cells exhibited peak CAR-T expansion by day 14, followed by a contraction phase, accompanied by a pronounced reduction in both the proportion and absolute count of circulating B cells. Notably, no cases of GvHD, CRS, ICANS, or other serious adverse events were observed (51). Together, these findings solidify the role of CD19-targeted CAR-T therapy as a transformative intervention for refractory SLE.

A promising therapeutic approach involves the isolation of adequate T cell populations from late-stage SLE patients, then genetic modification and autologous reinfusion (52). In a refractory SLE cohort (n=5; 4F/1M), autologous T cells transduced with a lentiviral anti-CD19 CAR vector and given after lymphodepletion (fludarabine/cyclophosphamide) achieved remission at 3 months (median SLEDAI = 0) with durable, drug-free remission beyond eight months; Naïve, non-class-switched B cells re-emerged at a median of 110 days; therapy was well tolerated with only mild, manageable CRS (36). Hu19-CD828Z is a second-generation CAR-T construct targeting CD19 with a fully human anti-CD19 antibody and a CD8α hinge/transmembrane (H/TM) domain fused to CD28 and CD3ζ signaling; in five SLE patients, Hu19-CD828Z–derived CAR-T cells showed CD19-dependent proliferation with markedly low release of inflammatory cytokines IL-2, IL-6, and IL-1β (37, 38). In a Phase I/II open-label, single-arm multicenter study of YTB323 (NCT05798117) in SLE (n=3 sentinel cohort), 12.5 × 10^6^ YTB323 CAR-T cells given at 28-day intervals depleted transient T cells and persistently depleted B cells, with marked reductions in SLEDAI and PhGA and declines in dsDNA, complement, and proteinuria; one grade 1 CMV reactivation and mild CRS occurred, both manageable with tocilizumab (53).

**Figure 4:**
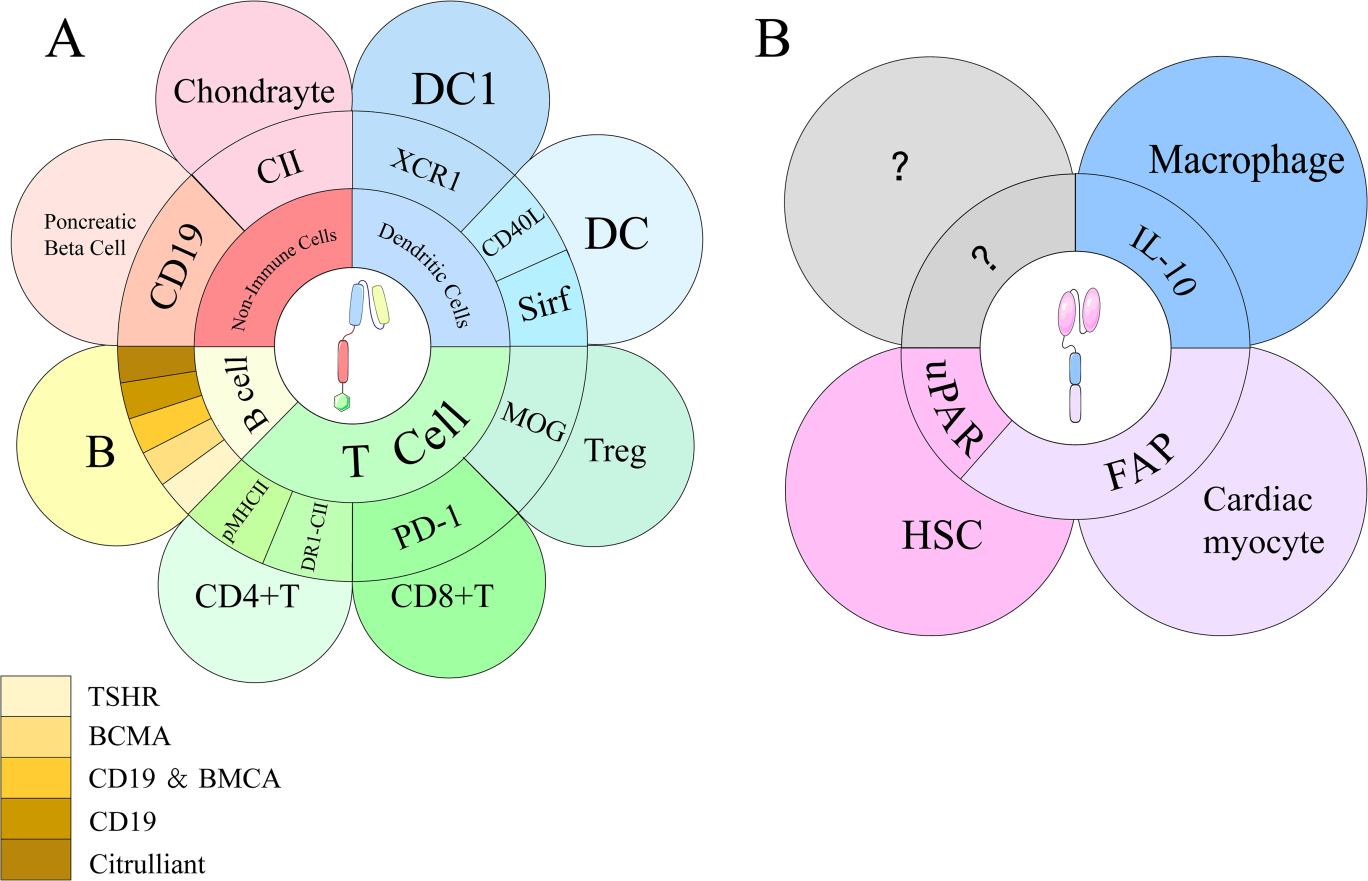
Targeted antigens of CAR-T and CAR-M on their respective target cells. **(A)** CAR-T cells engage with multiple immune and non-immune cell types, including T cells, B cells, dendritic cells (DCs), and chondrocytes. Key targets include PD-1, pMHC II, DR1-CII, XCR1, and autoantigens such as CII and citrullinated peptides. B cell targets involve CD19, BCMA, TSHR, and mAb287. **(B)** CAR-M cells interact with various cell types, including hepatic stellate cells (HSCs), cardiac myocytes, and macrophages, through distinct binding sites. Key targets include uPAR on HSCs, FAP on cardiac myocytes, and IL-10 on macrophages.

Emerging therapeutic strategies for SLE extend beyond B cell depletion to target CD4 follicular helper T (Tfh) cells: E4BP4 inhibits Tfh differentiation by repressing BCL-6 transcription via recruitment of HDAC1 and EZH2, while impaired E4BP4 function and IFN-I–driven IL-21/IFN-γ production promote Tfh expansion and autoreactive B cell responses, supporting Tfh-directed CAR-T approaches (54, 55). Peripheral blood analysis of 29 SLE patients shows significantly increased circulating Tfh-like cells versus healthy controls (p < 0.05); Tfh-like cells drive B cell differentiation into IgG-secreting plasma cells, with frequency correlating with circulating plasmablasts, dsDNA, and antinuclear antibody (ANA) levels (56). There is growing anticipation for CAR-T therapies targeting Tfh cells based on their physiological roles (**Figure 4**).

### 4.3 Sjögren’s syndrome

SS is a chronic AID primarily affecting the lacrimal and salivary glands, resulting in lymphocytic infiltration, exocrine gland damage, xerostomia, and xerophthalmia, with a significant female predominance (57, 58). Treatment options for SS remain limited. Its pathology involves the activation of B cells by T cells, leading to the secretion of inflammatory factors, immunoglobulins, and autoantibodies (anti-SSA and anti-SSB), resulting in inflammation, tissue damage, and clinical symptoms. B cells, surrounded by T cells within ectopic germinal centers, increase the risk of lymphoma (59, 60).

CAR-T therapy shows promise for SS, illustrated by a case where anti-CD19 CAR-T induced serologic remission (ANA and anti-Ro-52 negative by day 90) with SS Activity Index (SSDAI)SSDAI dropping from 5 to 2 and normalization of serum cytokines by six months, despite initial grade 2 cytokine release syndrome and grade 1 neurotoxicity; yet clinical data in SS remain scarce, underscoring the need for further research (9).

### 4.4 Myasthenia gravis and necrotizing myopathy

Myasthenia gravis (MG) is a T-cell-dependent, B-cell-mediated AID characterized by anti-acetylcholine receptor (AChR) antibodies, which cause muscle weakness and fatigue (61). Bispecifically targeted CAR T therapy is an emerging approach for MG that mitigates escape variants and lowers disease recurrence. In a notable case, a 64 year old man received lymphodepleting chemotherapy with cyclophosphamide (300 mg/m²) and fludarabine (30 mg/m²) prior to infusion of 1 × 10^6^ bispecific BCMA/CD19 targeted CAR T cells per kilogram. Over a total follow up of 210 days, early improvements were observed by day 14, with the Quantitative Myasthenia Gravis (QMG) score decreasing from 8 to 6 and the MG Activities of Daily Living (MG-ADL) score from 6 to 5; both scores subsequently reached 0 by day 60, indicating remission. Mild adverse events, including conjunctivitis and upper respiratory tract infections, occurred but resolved promptly (62, 63). In RNA-based CAR T therapy (rCAR T), CAR expression is achieved with messenger RNA (mRNA), exploiting its transient and non-replicating nature to yield predictable pharmacokinetics and enhanced safety. In a study of 14 MG patients receiving rCART, two achieved independence from intravenous immunoglobulin and three experienced mild side effects; over 6–12 months of follow up, gains were maintained when infusions were delivered across six weeks (64). In refractory MG treated with anti BCMA CAR T (NCT04561557), B cells returned to baseline around 18 months, with ~80% of reconstituted cells immature and a naïve repertoire, suggesting long term humoral suppression and relapse prevention (65). In immune-mediated necrotizing myopathy (IMNM), expanded CD8 T cells markedly reduce autoreactive CD4 T cells via enhanced cytotoxicity (66); in antisynthetase syndrome treated with CD19-targeted CAR T cells, declines in disease-associated serum markers (including anti Jo 1) and peripheral B cells were observed, with a transient CAR T peak on day 7 and a drop by day 14, suggesting limited durability of response; preconditioning included fludarabine and cyclophosphamide in the days preceding CAR T infusion, and MRI demonstrated radiological improvement post-treatment. However, CAR-T cell levels peaked on day 7 and rapidly decreased by day 14, suggesting that the therapeutic effect was not sustained (67).

### 4.5 Autoimmune encephalomyelitis and multiple sclerosis

Experimental autoimmune encephalomyelitis (EAE) is a CD4^+^ T cell-mediated AID characterized by central nervous system (CNS) demyelination, exhibiting pathology that resembles multiple sclerosis (MS), in which MΦs predominantly polarize to the M1 (68). Myelin oligodendrocyte glycoprotein (MOG) pMHCII CAR-T cells specifically target peptide-reactive T cells, with the affinity of the pMHCII TCR influencing their ability to recognize and eliminate target cells. Transduction of the pMHCII-CAR retrovirus is performed on initial CD8 T cells activated with anti-CD3 and anti-CD28. In host mice, pMHCII CAR-T cells exhibit high-affinity TCR specificity, effectively eliminating both initial and activated CD4 T cells, thereby preventing the onset of EAE. In contrast to foreign reactive TCRs, the self-reactive TCRs encountered in the thymus typically have lower affinity, and notably, these lower-affinity CD4 T cells dominate during EAE. To improve sensitivity to low-affinity TCRs and reverse established EAE, artificial disulfide bond trapping (DST) and inhibition of Fas function have been integrated into the structure of pMHCII CARs (69). MOG-CAR-T regulatory cells (Tregs) suppress autoreactive T cells, selectively migrate to the CNS, and reduce pathogenic activity, thereby delaying the onset of EAE (70). After cyclophosphamide preconditioning, anti-CD19 CAR-T cell therapy induced peripheral and CNS B cell depletion, reduced clinical scores and lymphocyte infiltration, and ameliorated EAE (71). In Dendritic cell (DC) type 1 (DC1)-driven autoimmunity, targeting X-C motif chemokine receptor 1 (XCR1) CAR T cells deplete DC1 across multiple organs in mouse models (including inguinal and cervical lymph nodes, spleen, lung, and liver) and inhibit Th1 driven EAE progression; DC1 depletion increases DC2 without altering total dendritic cells; CD4 XCR1 CAR T cells produce higher pro inflammatory cytokines (TNF α, IL 27, IL 10) than CD8 XCR1 CAR T cells, indicating higher CRS risk, and the approach remains largely proof of concept requiring further safety evaluation (72).

### 4.6 Neuromyelitis optica spectrum disorder

In neuromyelitis optica spectrum disease (NMOSD), anti BCMA CAR T therapy demonstrates therapeutic potential, with engineered CAR T cells showing enhanced chemotaxis and the ability to traverse both the blood–brain barrier and CSF to target plasma cells and plasmablasts, thereby attenuating neuroinflammation (73). In a clinical study, twelve patients (10 females; median age 49.5 years, range 30–67) received anti BCMA CAR T infusions. Over a median follow up of 5.5 months, 11 patients remained relapse free and all reported improvements in disability and quality of life. Serum aquaporin-4 (AQP-4) antibody levels trended downward in 11 patients at baseline. At final follow up, EDSS scores decreased for all patients compared with baseline. All patients experienced grade 1–2 cytokine release syndrome and leukopenia, most of which resolved within four weeks. Overall, anti BCMA CAR T therapy demonstrated tolerable safety and potential efficacy in relapsed/refractory AQP4 IgG seropositive NMOSD (74).

### 4.7 Type 1 diabetes

Type 1 diabetes (T1D) is an organ-specific AID characterized by the CD4 T cell-mediated destruction of pancreatic β cells (75). The B:9–23 peptide binds to the non-obese diabetic (NOD) MHC II molecule (I-Ag7), forming a complex that stimulates pathogenic B:9-23-specific CD4 T cells. Zhang et al. developed a monoclonal antibody (mAb287) targeting this critical I-Ag7-B:9-23(R3) complex. A single infusion of CAR-T cells has been shown to delay T1D onset in NOD mice. Compared to untreated animals, a single infusion of 287-CAR CD8 T cells into young (5 weeks old) NOD mice significantly delayed the onset of overt hyperglycemia (p = 0.022). However, the protective effect diminished over time, with no significant difference in overall incidence rates between the 287-CAR CD8 T cell therapy group and the control group at 30 weeks (76).

Depletion of regulatory T cells (Tregs) disrupts T cell homeostasis and can lead to T1D. Strategies to repair or replace Tregs may reverse autoimmunity and protect β cells. Attempts to generate HPi2-directed CAR-Tregs using anti-HPi2 scFv failed because the antibody was broadly expressed on CD4 T cells, rendering the approach non-viable (77). In a Phase I trial, 14 adults with T1D received *ex vivo*–expanded autologous Tregs (0.05 × 10^8^ to 26 × 10^8^) across four dose cohorts, with peak circulating Tregs reaching up to 25% at one year and no infusion reactions or high-grade adverse events. These results support a Phase II trial to evaluate Treg therapy (78), and insulin-specific CAR-Tregs remain a promising avenue for immunomodulation in T1D (79), potentially enhanced by combining mAb287 CAR-T cells with specific CAR-Tregs.

### 4.8 Graves’ disease

Targeting the thyroid-stimulating hormone receptor (TSHR) constitutes a novel therapeutic strategy for Graves’ disease (GD), an AID driven by thyroid receptor antibodies (TRAb). Duan et al. (80) were the first to develop TSHR CAR-T cells by utilizing TSHR’s extracellular region in mouse experiments. Unlike traditional scFvs, TSHR is a G protein-coupled receptor with four extracellular domains that specifically bind pathogenic autoantibodies to its N-terminal (21–413 amino acids), offering a novel approach for treating Graves’ disease. TSHR-CAR-T cells effectively reduce levels of TRAb and increase the secretion of IL-2 and IFN-γ in a dose-dependent manner, although serum antibodies may interfere with CAR-T cell functionality, necessitating further exploration of affinity regulation or Fc receptor blockade. Cheever (81) proposed the chimeric autoantibody receptor (CAAR), where autoantigens act as binding domains for CAR-T cells. TSHR CAAR T cells, co-cultured *in vitro* with anti-TSHR B cells that were transduced by lentivirus, exhibited significant increases in IFNγ, IL-2, and TNF levels within 24 hours, effectively eliminating autoreactive anti-TSHR B cells without affecting other B cell populations. However, TSHR CAAR T cells showed reduced cytotoxicity against anti-TSHR B cells in the plasma of Graves’ disease patients compared to healthy plasma. These two experiments represent significant innovations in the field, as the novel scFv structure and the reformulated CAR-T approach provide new therapeutic directions for CAR-T cell therapy. Nonetheless, as both studies are preclinical, further evaluation of their efficacy and safety is essential through additional research.

### 4.9 Primary biliary cholangitis

PD-1, an immune checkpoint receptor on activated T and B cells, interacts with PD ligand 1 (PD-L1) to inhibit T cells (82). Impaired PD-1/PD-L1 signaling increases susceptibility to AID (83). Notably, combining PD-1 inhibition with CAR-T therapy is an established strategy, and directly targeting PD-1 represents a bold approach (84). In primary biliary cholangitis (PBC), PD-1-targeted CAR-T cells deplete CD4 T cells, reducing biliary epithelial cytotoxicity in mice (85). It is important to emphasize that treating AID by targeting PD-1 necessitates strict safety monitoring. Furthermore, several preclinical model experiments must be conducted to ensure the safety and efficacy of this approach.

### 4.10 Immune thrombocytopenia

Immune thrombocytopenia (ITP) is an AID characterized by anti-platelet autoantibodies, with glycoprotein Ibα (GPIbα) serving as a key autoantigen linked to refractoriness. In a modified mouse model, CD19 CAR-T cells targeting GPIbα-positive cells demonstrated accelerated platelet count recovery, effective depletion of CD19 B cells and CD138 plasma cells, and a significant reduction in anti-GPIbα autoantibodies in both *in vivo* and *in vitro* experiments. Additionally, CD19 CAR-T cell therapy notably alters T cell subsets by increasing populations of regulatory T cells, helper T cell 1, and helper T cell 17. Monitoring of body/spleen weight and body temperature indicated no significant CRS, suggesting favorable safety (86). Nonetheless, further clinical studies are required to assess the safety and efficacy of this approach in human patients.

### 4.11 Fibrosis and inflammation

CAR-M therapies show promise for treating fibrosis by targeting profibrotic cells and remodeling the extracellular matrix. A FAP-targeting CAR-M incorporating an anti-FAP scFv and the intracellular signaling domain CD147 was administered intravenously after cardiac ischemia–reperfusion (I/R) in mice, with echocardiographic improvements in left ventricular ejection fraction (LVEF) and fractional shortening (FS), reduced fibroblast numbers in the infarct area, and no observed cardiac toxicity (87). Intravenous delivery of a uPAR-targeting CAR-M in cirrhosis models markedly reduced serum ALT and fibrosis-associated gene expression (Col1a1, Col2a1, Acta2), accompanied by increased hepatocyte proliferation and partial liver function recovery; adaptive immune responses were suggested by enhanced CD8 T cell–mediated killing post-phagocytosis, without severe adverse events (88). FAP-CAR-Ms and FAP-CAR-ΔZETA-Ms derived from bone marrow inhibited activation of FAP-positive hepatic stellate cells *in vivo*, promoting extracellular matrix degradation via downregulation of Col1a1 and Acta2 and upregulation of MMP-9 and MMP-13 (89). A novel *in vivo* approach using mannose-modified mRNA lipid nanoparticles to target CD206 on M2 reprogrammed them into FAP-CAR-M, yielding decreases in activated cancer-associated fibroblast markers, collagen volume, and Col1a1 in a pancreatic ductal adenocarcinoma model (90). Collectively, these studies illustrate the therapeutic potential of CAR-M–mediated anti-fibrotic effects across organ systems, while underscoring the need for further investigation into *in vivo* delivery mechanisms, safety, and off-target risks to support clinical translation.

CAR-M therapies are expected to exert strong anti-inflammatory effects in intervertebral disc degeneration (IDD) by promoting clearance of apoptotic nucleus pulposus cells (NPCs) within the intervertebral disc (IVD) and modulating the inflammatory microenvironment (91). Zhou et al. developed a THP-1–derived CAR-engineered MΦs (CAR-eM) that upregulates brain-specific angiogenesis inhibitor 1 (BAI1) expression and employs an intradiscal annular microneedle delivery system to target deeper layers of the intervertebral disc (IVD) *in vitro* (92). The robust phagocytic activity of the MΦs facilitates clearance of apoptotic nucleus pulposus cells (apo-NPCs), improves the inflammatory milieu, and supports repair of damaged IVD tissue. In surgically resected nucleus pulposus specimens, the ratios of CD206/CD68 and CD86–expressing MΦs significantly increased, indicating a shift toward M2 polarization (92). Co-culture experiments involving tissue-derived macrophages (TDMs) and bone marrow–derived MΦs (BMDMs) with apo-NPCs, assessed by flow cytometry, revealed that efferocytosis can regulate the propensity for M2 polarization, a finding further validated at the transcriptional level (92).

## 5 CAR-T/M advantages and limitations in autoimmune diseases

### 5.1 Adaptation and advantage conditions

In order to facilitate a direct comparison of the therapeutic profiles of CAR-T and CAR-M therapies, key quantitative metrics were extracted from a selection of representative clinical studies (for CAR-T) and preclinical studies (for CAR-M). These are summarized in **Table 2**. It is imperative to exercise caution when interpreting these data, given the inherent disparities in research platforms and developmental stages. Nevertheless, these representative benchmarks offer valuable insights into the fundamental distinctions between the two modalities with respect to their mechanisms of action, durability of response, and safety profiles.

**Table 2:** Comparison of key characteristics between CAR-T and CAR-M therapies for autoimmune diseases.

Comparison metric	CAR-T cell therapy (3, 48, 51)	CAR-M cell therapy (8, 31)	Analysis and implications
Clinical Stage	Clinical validation (SLE)	Preclinical proof-of-concept (Arthritis model)	• This disparity defines a fundamental difference in developmental risk and translational pathways. • CAR-T is undergoing efficacy and safety verification, whereas CAR-M’s therapeutic potential and risks remain largely unexplored.
Core Mechanism	Cytotoxic depletion of immune cells (Subtractive)	Microenvironment remodeling via phagocytosis/cytokines (Regulatory)	• The fundamental mechanistic divergence—cytotoxic depletion versus regulatory remodeling—dictates distinct therapeutic applications and risk profiles.
Key Efficacy Outcome	SLEDAI=0; Anti-dsDNA seroconversion	70% reduction in clinical score	• The efficacy in distinct disease models suggests complementary therapeutic niches: CAR-T for systemic humoral autoimmunity and CAR-M for localized, macrophage-driven pathologies.
Cellular Persistence	Long-term (>8 months)	Short-term (30 days)	• The disparity in durability underscores a critical challenge for CAR-M as a short-lived intervention, potentially requiring repeat dosing and complicating its clinical translation for chronic diseases.
Primary Safety Event	Manageable CRS (G1-2)	No severe toxicity reported (preclinical)	• The CRS associated with CAR-T is a known, manageable risk of its potent mechanism. • The absence of reported severe toxicity for CAR-M reflects the limitations of preclinical research; its potential long-term tissue impacts remain a primary safety concern.

Data are derived from representative clinical studies in lupus (CAR-T) and preclinical models of arthritis (CAR-M). The fundamental distinctions in clinical maturity, mechanism of action, efficacy, persistence, and safety profiles are summarized. The mechanistic divergence—subtractive immune cell depletion (CAR-T) versus regulatory microenvironment remodeling (CAR-M)—dictates their respective application scenarios: systemic humoral immunity versus organ-specific macrophage-driven pathology. The significant difference in cellular persistence underscores a key translational challenge for CAR-M.

Mechanistic definitions: Subtractive: Depletion of specific immune cell populations via cytotoxicity; Regulatory: Remodeling of the tissue microenvironment via phagocytosis and cytokine secretion.

Compared with the emerging CAR-M therapy, CAR-T cell therapy has a larger clinical sample size and demonstrates favorable safety and efficacy across AID (93), including effective B cell ablation, seroconversion, improved disease activity, and durable remission at six months. CD19 CAR-T cell therapy demonstrated a favorable safety profile and was generally well-tolerated in patients with severe AID. In a 15-patient study with severe autoimmune diseases, all achieved symptomatic remission after a median 15-month follow-up, with a mean B-cell aplasia duration of 112 days and a manageable safety profile that was primarily characterized by grade 1 CRS (94). However, rare cases of low-grade CRS, ICANS, and prolonged cytopenia have been reported, predominantly in patients with coexisting hematologic malignancies (95).

CAR-T cell therapy demonstrates superior cytotoxicity, efficacy, and durability compared to treatments involving monoclonal antibodies (mAbs). CAR-T enables more extensive depletion of B cells compared to mAb treatment, effectively targeting B cells in both the CNS (71) and resident tissues (96). Anti-CD20 mAbs, such as rituximab, primarily deplete peripheral B cells, leaving CNS B cells unaffected, with regeneration occurring within 6 to 12 months, thereby increasing the risk of disease relapse (97). A similar limitation was observed with the anti-CD19 monoclonal antibody obexelimab in a Phase II trial for SLE. In this trial, patients received intravenous injections of obexelimab at a dose of 5 mg/kg biweekly until week 32 or until loss of improvement (LOI) was observed. Analysis revealed a 50% reduction in B cell levels; while the time to LOI increased, the primary endpoint was not achieved (98). It is noteworthy that anti-BCMA CAR-T targeted therapy has a more pronounced effect on the characteristics of individual autoantibodies (99). CD19 is recognized as one of the most reliable surface biomarkers for B cells, with expression initiated in pre-B cells and continuing until the terminal differentiation of these cells into plasma cells (100). Conversely, BCMA is predominantly expressed in specific subsets at the late stages of B cell differentiation, including plasma cells, plasmablasts, certain memory B cells, and malignant B cells (e.g., malignant plasma cells in multiple myeloma) (101). Bodansky et al. compared the effects of three major B cell depletion therapies—rituximab (anti-CD20), anti-CD19 CAR-T cells, and anti-BCMA CAR-T cells—using PhIP-Seq analysis. A comparison of the PhIP-Seq enrichment profiles from nine individuals, obtained before and after anti-BCMA CAR-T cell treatment, revealed minimal similarity in the autoreactive repertoire post-treatment (median Pearson’s r value = 0.006; Q1 = 0.002 and Q3 = 0.130 for 8 out of 9 individuals). This complete “reset” of the autoreactive repertoire indicates that successful treatment with anti-BCMA CAR-T cells is sufficient to eliminate antibody-producing plasma cells accumulated over a lifetime (52). Müller conducted a comparison of CD19 and BCMA in the context of AID. A 45-year-old female patient with refractory Jo-1 associated anti-synthetase syndrome experienced a relapse nine months after initially successful CD19-CAR-T cell therapy. Upon reinfusion with the same product, CAR-T cell expansion failed, and anti-CD19 CAR-T cells were detected. After thorough evaluation, the patient received BCMA-CAR-T cell therapy, which resulted in successful CAR-T cell expansion, clearance of plasma cells in lymphoid tissues, decreased autoantibody levels, and the reinduction of stable drug-free remission. However, further evaluation of safety and efficacy is required (102). CAR-T cells demonstrate greater durability and potential efficacy than monoclonal antibodies, owing to their proliferative and persistent nature. In two patients with chronic lymphocytic leukemia, persistently active CD19 CAR-T cells achieved complete remission (CR) after infusion, with detectable CAR-T cells maintained for more than a decade while remission persisted. At later time points, the CAR-T cell pool was predominantly comprised of highly activated CD4 T cells. Single-cell analyses revealed that these long-lived CD4 CAR-T cells possessed cytotoxic features alongside sustained functional activation and proliferation (14). However, due to their long-term persistence, the safety of these treatments can be more challenging to manage, particularly concerning cytokine release CRS and ICANS.

The inherent advantages of CAR-M for AID are primarily reflected in their high infiltration capacity into the ECM and their low interference with the immune microenvironment (103, 104). In contrast, CAR-T cells exhibit limited infiltration capacity into the ECM. Mondal et al. developed fucosylated sLe(X)-modified CAR-T cells that possess enhanced E-selectin binding properties, leading to a 10-fold increase in infiltration efficiency into the bone marrow compared to non-fucosylated CAR-T cells (105). Additionally, CAR-M cells have a limited lifespan *in vivo*, which reduces the risk of CRS, neurotoxicity, and GvHD. In a clinical trial, tumor patients treated with autologous CAR-M demonstrated only transient cytokine fluctuations and lymphocytopenia, with no other high-grade (≥3) adverse events or CRS observed (106). However, this short survival time can also be a double-edged sword, as it limits the therapeutic effect (29). Furthermore, although infused MΦs have the capacity to infiltrate extensively, most CAR-M tend to localize in organs such as the liver and lungs, which may limit their overall numbers and therapeutic efficacy (107).

In stark contrast to the advancing clinical trajectory of CAR-T therapy, CAR-M therapy remains firmly entrenched in the preclinical proof-of-concept stage for AID applications. Despite compelling preclinical evidence for fibrosis resolution and anti-inflammatory effects, formidable translational barriers—including the limited persistence (29), hepatic sequestration (107), and host immune clearance (108) of adoptively transferred macrophages—must be overcome before the clinical potential of CAR-M can be realistically assessed.

### 5.2 Limitations and perspectives

The clinical translation of CAR therapy in AID faces significant practical hurdles of cost and manufacturing complexity. The autologous process, requiring patient-specific T cell isolation, modification, and expansion, remains resource-intensive and economically challenging, potentially limiting widespread application in AID populations (109, 110). In the AID context, where repeated or urgent treatment may be necessary, rapid and cost-effective production is paramount. Nanocarrier technology offers a promising solution for both CAR-T and CAR-M production, potentially streamlining manufacturing and reducing costs (111). For instance, *in vivo* reprogramming via nanocarriers could bypass resource-intensive *ex vivo* processes, enabling quicker and more accessible therapy for AID patients (32). Furthermore, while cryopreservation can reduce T cell viability, optimized protocols still yield sufficient cells for CAR-T production, addressing logistical concerns in AID treatment centers (112). Safety of genetic modification is also critical; vector design, such as the third-generation KL-h198a28z with its enhanced safety profile, requires careful consideration for chronic AID applications where long-term safety is a primary concern (113).

Innovations in CAR-M delivery hold particular promise for treating organ-specific AID. Inhalable CARmRNA@aCD206 sEVs carry CAR-mRNA with surface-integrated anti-CD206 scFvs to enable *in situ* CAR-M generation in the lung; inhaled CARmRNA@aCD206 sEVs accumulate in lung tissue and selectively deliver CAR-mRNA to MΦs, promoting local CAR-M production and presenting a promising immunotherapy strategy (114). Chuang et al. describe a nano-immunoengineering approach combining THP-1–derived CD47 CAR-M with surface-anchored HPβ-CD lipid nanoparticles (β-CD LNPs); under oxidative stress, HPβ-CD released from β-CD LNPs dissolves cholesterol crystals and upregulates the liver X receptor (LXR) pathway in MΦs, enhancing clearance of apoptotic debris, with THP-1 MΦs showing 1.57-fold increases in Mertk and IL-10 compared with untreated MΦs (115). By fusing a humanized single-chain variable fragment with FcγRIIa and integrating short hairpin RNA, SIRPα can be silenced, disrupting CD47–SIRPα signaling; THP-1–derived CAR-shSIRPα-M exhibit high levels of CD80, CD86, and TNF-α under resting conditions, with significant upregulation of M1-associated and glycolysis-related genes, suggesting CAR modification may bolster M1 polarization via enhanced glycolysis (116). Similarly, CAR-M strategies that incorporate anti-inflammatory intracellular domains (e.g., IL-4Rα) have demonstrated efficacy in reducing inflammation in a renal injury model (117), suggesting a direct application in autoimmune nephritis. Despite these advances, CAR-M research in AIDs remains largely preclinical, underscoring the need for clinical safety validation, and systemic CAR-M distribution, notably hepatic accumulation, poses risks of off-target toxicity and reduced efficacy (108). Structural modifications in CAR receptor design signify significant progress in the field. Innovative extracellular domain designs, such as TSHR-CAR-T cells, have emerged (80). Furthermore, intracellular domains, including Megf10, FcRγ, and PI3KP85, have been shown to enhance phagocytosis, with tandem structures amplifying this effect (6). Their efficacy and safety have been validated in humanized mouse models (7). The intracellular domain TIRs can induce stronger activation, which may open new avenues for applications in non-tumor diseases such as AID (30). Additionally, rCAR-T cells signify a pioneering advancement in immunotherapy, overcoming the limitations of conventional DNA-based CAR-T, which relies on DNA proliferation for CAR signal amplification and is associated with unpredictable pharmacokinetics and severe adverse events, particularly CRS (64). Nevertheless, current research remains limited, necessitating further validation of efficacy.

CAR-T cell source and culture duration determine efficacy and safety across oncology and AID, with shorter *ex vivo* culture yielding less differentiated CAR-T cells that exhibit enhanced effector function and proliferative capacity, especially beneficial for sustained immune regulation in AID (118). AutoCAR-T cells prevent immune rejection, while allogeneic T cells from healthy donors exhibit stronger cytotoxicity and allow for quicker treatment through pre-collection. Second-generation anti-CD19 CAR-T cells (KYV-101) were generated from 20 patients’ PBMCs, enriched for CD4/CD8 T cells, transduced with KL-h198a28z lentivirus, and expanded *in vitro*, achieving CAR expression across neurological, rheumatic, and healthy-donor groups (47–77%, 37–73%, 50–75% respectively) with 11–66 fold expansion by day 8, indicating comparable efficacy across disease types (119). The choice between autologous and allogeneic CAR-T cell sources involves a critical risk-benefit calculation in AID. Allogeneic ‘off-the-shelf’ products allow for rapid treatment initiation—a significant advantage in rapidly progressive AID. Their potent cytotoxicity, as seen in studies like the BCP-ALL trial (120), could translate to more profound B-cell depletion in AID conditions. However, this potency comes with risks: the high proportion of naïve T cells in healthy donors may elevate the risk of severe CRS and ICANS, and alloreactive T cells pose a threat of GvHD (120, 121).

Toxicity management strategies for CRS and ICANS in oncology may need adaptation for AID, relying on supportive care, tocilizumab, and corticosteroids, and Anti-CD19 CAR-T–driven rises in IL-6, IL-10, and IFN-γ promote perforin/gasdermin release that amplifies tissue damage and sHLH/MAS and CRS (122–124). In the CTL019 cohort (n = 45; 24 male, 21 female; median age 12), the median time from CRS onset to first tocilizumab dose was 4 days (0–18). CRS resolution within 14 days after the first tocilizumab dose occurred in 69% (31/45; 95% CI 53–82%), with up to two doses and fever resolution for ≥24 hours without vasopressors or non-corticosteroid treatments. Further studies are needed to define optimal tocilizumab dosing and safety for CAR-T–induced CRS (125). For tocilizumab-resistant CRS, corticosteroids are used, but higher cumulative doses and early/prolonged use after CAR-T infusion are linked to shorter overall survival (126). Exploring AID-specific toxicity mitigation strategies, such as concurrent AIM2 inflammasome blockade, is an important future direction (127). In AID, where sustained immune modulation is often prioritised over short-term ablation, proactive management of acute cell therapy-related risks is essential. This has driven the development of next-generation safety strategies that extend beyond conventional interventions such as tocilizumab and corticosteroids to enable preemptive control of CAR-T cell activity. The following approaches have been identified: The following examples are provided of switches that ensure the safety of the system: inducible caspase-9 suicide genes for emergency cell ablation (128), CRISPR-Cas9-mediated disruption of TCR expression to reduce graft-versus-host disease risk in allogeneic products (129, 130), logic-gated CAR circuits that require sensing of complex, disease-specific signals to trigger activation (131), and transient expression systems employing non-viral mRNA delivery to restrict CAR persistence (132), thereby precisely defining the therapeutic window. Despite the evident potential of these innovations, it is crucial to acknowledge the paucity of safety data specifically pertaining to AID populations (133). This underscores the imperative for additional clinical validation within this specific context.

A paramount challenge in achieving long-term remission in AID is combating CAR-T-cell exhaustion and ensuring persistent regulatory function, which requires sustained immune regulation. Mechanisms driving exhaustion, such as oxidative stress (134, 135), present key targets. A previous study demonstrated that during primary viral infection, counteracting ROS effects with antioxidants reduced T cell expansion (136). Mitochondria-targeted antioxidant treatments, such as mitoquinone and MitoTEMPO, mitigate oxidative stress from mitochondrial membrane potential (ΔΨm) depolarization and excessive ROS levels in exhausted CD4 T cells, significantly enhancing cell viability and antiviral function (137). Additionally, IL-21 treatment of CAR-T cells promotes oxidative phosphorylation (OXPHOS) and reduces levels of HIF1A, CD38, SIRT1, TET2, CTLA4, and TOX2, suggesting that IL-21 may inhibit T cell exhaustion and senescence through the CD38-NAD-SIRT1 axis (138). Conversely, the limited proliferative capacity and persistence of CAR-M (139, 140) is a major hurdle for sustained antigen clearance in chronic AID. Developing strategies to enhance CAR-M survival and function is therefore a critical research priority for AID applications.

The polarization state of CAR-M can be regulated by the intracellular domain (141). In anti-tumor therapy, enhancing therapeutic effects through M1 polarization of CAR-M has proven effective, as demonstrated with the tandem CD3ζ-TIR dual-signal CAR-M (30). In non-tumor diseases, M1 polarization may be detrimental. When MΦs fail to maintain a proper balance during the wound healing process—such as not transitioning from the M1 to the M2 phenotype—it can impair wound healing (142). Chronic wound formation results in sustained inflammation that hinders tissue repair (143). A reduced phagocytic ability of MΦs toward apoptotic neutrophils leads to the accumulation of inflammatory substances, obstructing the transition to the M2b phenotype, thereby exacerbating inflammation and delaying the healing process (144). Exosomes rich in miR-146a-5p can inhibit the activation of M1 and reduce the expression and release of pro-inflammatory factors, such as MCP-1, IL-6, and TNF-α, by targeting CD80 (145). Conversely, promoting M2 polarization can be beneficial by resolving inflammation and promoting tissue repair, as seen in liver fibrosis and neuroinflammation models (146, 147). The critical challenge for AID therapy is designing CAR-Ms whose polarization state (e.g., towards pro-resolving M2a or M2c phenotypes (148)]) can be tuned to the disease context—suppressing inflammation without hindering essential repair mechanisms. The safety and efficacy of CAR-M in AID will be profoundly influenced by achieving this delicate balance, necessitating extensive preclinical evaluation in specific AID models.

The employment of combination strategies that capitalise on the strengths of both CAR-T and CAR-M cells represents a promising new frontier in the treatment of AID. Two synergistic models are envisioned: a sequential approach, where CAR-Ms first precondition the microenvironment by clearing antigenic debris and immune complexes to enable safer, more effective CAR-T deployment against pathogenic lymphocytes; or co-administration, simultaneously targeting both the inflammatory milieu (via CAR-M) and adaptive immune drivers (via CAR-T) for a rapid, comprehensive reset (104, 149). The distinctive capacity of CAR-M to target a more extensive array of entities serves to further expand its potential. Future research should concentrate on adapting these synergistic regimens to the specific pathophysiology of individual AIDs, thereby creating the basis for personalised cellular therapies.

Beyond the biological and clinical limitations discussed above, the successful translation of CAR-based therapies for autoimmune diseases must also address profound manufacturing, accessibility, and ethical challenges (10, 11). The autologous process remains prohibitively expensive and logistically complex, limiting patient access globally (109, 110). While allogeneic ‘off-the-shelf’ products offer a solution, they require sophisticated gene editing to mitigate graft-versus-host disease risks, raising additional safety and ethical considerations (120, 121). Emerging technologies, particularly *in vivo* reprogramming via non-viral delivery platforms like lipid nanoparticles, hold the promise of radically simplifying manufacturing, reducing costs, and enabling rapid and repeat dosing (32, 33, 111). However, these approaches necessitate careful ethical scrutiny regarding long-term genomic safety and equitable implementation (113). Therefore, the future of CAR therapy in autoimmunity depends not only on scientific innovation but also on parallel advancements in affordable manufacturing, thoughtful health policy, and ethical oversight to ensure these transformative treatments can reach the broad patient population in need (10, 11, 109).

## 6 Conclusion

In the future, the clinical translation of CAR therapies for autoimmune diseases will require a strategic shift towards overcoming three fundamental barriers. Firstly, the development of dual-antigen sensing CARs to achieve disease-lesion-specific targeting. Secondly, the engineering of CAR-M with enhanced tissue-penetrating and extracellular matrix remodeling capabilities to surmount fibrotic barriers. Thirdly, the adoption of transient *in vivo* mRNA delivery platforms to ensure controllable persistence and mitigate safety risks. By focusing on these targeted strategies—smarter targeting, better delivery, and safer control—the next generation of cellular therapies can be tailored to meet the chronic and complex demands of autoimmune pathologies.

## References

[1] Schett G Mackensen A Mougiakakos D . Car T-cell therapy in autoimmune diseases. Lancet (London England). (2023) 402:2034–44. doi: 10.1016/s0140-6736(23)01126-1, PMID: 37748491

[2] Gross G Waks T Eshhar Z . Expression of immunoglobulin-T-cell receptor chimeric molecules as functional receptors with antibody-type specificity. Proc Natl Acad Sci U S A. (1989) 86:10024–8. doi: 10.1073/pnas.86.24.10024, PMID: 2513569 PMC298636

[3] Mougiakakos D Krönke G Völkl S Kretschmann S Aigner M Kharboutli S . Cd19-targeted car T cells in refractory systemic lupus erythematosus. New Engl J Med. (2021) 385:567–9. doi: 10.1056/NEJMc2107725, PMID: 34347960

[4] Zugasti I Espinosa-Aroca L Fidyt K Mulens-Arias V Diaz-Beya M Juan M . Car-T cell therapy for cancer: current challenges and future directions. Signal transduction targeted Ther. (2025) 10:210. doi: 10.1038/s41392-025-02269-w, PMID: 40610404 PMC12229403

[5] Lin H Cheng J Mu W Zhou J Zhu L . Advances in universal Car-T cell therapy. Front Immunol. (2021) 12:744823. doi: 10.3389/fimmu.2021.744823, PMID: 34691052 PMC8526896

[6] Morrissey MA Williamson AP Steinbach AM Roberts EW Kern N Headley MB . Chimeric antigen receptors that trigger phagocytosis. eLife. (2018) 7:1–21. doi: 10.7554/eLife.36688, PMID: 29862966 PMC6008046

[7] Klichinsky M Ruella M Shestova O Lu XM Best A Zeeman M . Human chimeric antigen receptor macrophages for cancer immunotherapy. Nat Biotechnol. (2020) 38:947–53. doi: 10.1038/s41587-020-0462-y, PMID: 32361713 PMC7883632

[8] Huang Y Wang Z Zhong C Chen H Chen X Cao C . Crispra engineered elite macrophages enable adoptive cell therapy for rheumatoid arthritis. Innov Med. (2024) 2:100050. doi: 10.59717/j.xinn-med.2024.100050

[9] Sheng L Zhang Y Song Q Jiang X Cao W Li L . Concurrent remission of lymphoma and Sjögren’s disease following anti-Cd19 chimeric antigen receptor-T cell therapy for diffuse large B-cell lymphoma: A case report. Front Immunol. (2023) 14:1298815. doi: 10.3389/fimmu.2023.1298815, PMID: 38173731 PMC10762793

[10] Chohan KL Siegler EL Kenderian SS . Car-T cell therapy: the efficacy and toxicity balance. Curr hematologic Malignancy Rep. (2023) 18:9–18. doi: 10.1007/s11899-023-00687-7, PMID: 36763238 PMC10505056

[11] Brudno JN Kochenderfer JN . Current understanding and management of Car T cell-associated toxicities. Nat Rev Clin Oncol. (2024) 21:501–21. doi: 10.1038/s41571-024-00903-0, PMID: 38769449 PMC11529341

[12] Levine BL Miskin J Wonnacott K Keir C . Global manufacturing of Car T cell therapy. Mol Ther Methods Clin Dev. (2017) 4:92–101. doi: 10.1016/j.omtm.2016.12.006, PMID: 28344995 PMC5363291

[13] Wang X Borquez-Ojeda O Stefanski J Du F Qu J Chaudhari J . Depletion of high-content Cd14(+) cells from apheresis products is critical for successful transduction and expansion of car T cells during large-scale Cgmp manufacturing. Mol Ther Methods Clin Dev. (2021) 22:377–87. doi: 10.1016/j.omtm.2021.06.014, PMID: 34514029 PMC8411225

[14] Melenhorst JJ Chen GM Wang M Porter DL Chen C Collins MA . Decade-long leukaemia remissions with persistence of Cd4(+) Car T cells. Nature. (2022) 602:503–9. doi: 10.1038/s41586-021-04390-6, PMID: 35110735 PMC9166916

[15] Arcangeli S Falcone L Camisa B De Girardi F Biondi M Giglio F . Next-generation manufacturing protocols enriching T(Scm) Car T cells can overcome disease-specific T cell defects in cancer patients. Front Immunol. (2020) 11:1217. doi: 10.3389/fimmu.2020.01217, PMID: 32636841 PMC7317024

[16] Hou R Zhang X Wang X Zhao X Li S Guan Z . *In vivo* manufacture and manipulation of Car-T cells for better druggability. Cancer metastasis Rev. (2024) 43:1075–93. doi: 10.1007/s10555-024-10185-8, PMID: 38592427

[17] Ayala Ceja M Khericha M Harris CM Puig-Saus C Chen YY . Car-T cell manufacturing: major process parameters and next-generation strategies. J Exp Med. (2024) 221:1–14. doi: 10.1084/jem.20230903, PMID: 38226974 PMC10791545

[18] Jandova M Stacey GN Lanska M Gregor I Rozsivalova P Bekova L . The role of cryopreservation techniques in manufacturing, transport, and storage of Car-T therapy products. Cryo Lett. (2023) 44:123–33. doi: 10.54680/fr23310110112 37883165

[19] Eshhar Z Waks T Oren T Berke G Kaufmann Y . Cytotoxic T cell hybridomas: generation and characterization. Curr topics Microbiol Immunol. (1982) 100:11–8. doi: 10.1007/978-3-642-68586-6_2, PMID: 6980088

[20] Chmielewski M Abken H . Trucks: the fourth generation of cars. Expert Opin Biol Ther. (2015) 15:1145–54. doi: 10.1517/14712598.2015.1046430, PMID: 25985798

[21] Funes SC Rios M Escobar-Vera J Kalergis AM . Implications of macrophage polarization in autoimmunity. Immunology. (2018) 154:186–95. doi: 10.1111/imm.12910, PMID: 29455468 PMC5980179

[22] Kagoya Y Tanaka S Guo T Anczurowski M Wang CH Saso K . A novel chimeric antigen receptor containing a Jak-Stat signaling domain mediates superior antitumor effects. Nat Med. (2018) 24:352–9. doi: 10.1038/nm.4478, PMID: 29400710 PMC5839992

[23] Fan M Zheng J Huang Y Lu M Shang Z Du M . Nanoparticle-mediated universal Car-T therapy. Int J pharmaceutics. (2024) 666:124779. doi: 10.1016/j.ijpharm.2024.124779, PMID: 39349228

[24] Colin S Chinetti-Gbaguidi G Staels B . Macrophage phenotypes in atherosclerosis. Immunol Rev. (2014) 262:153–66. doi: 10.1111/imr.12218, PMID: 25319333

[25] Hadiloo K Taremi S Heidari M Esmaeilzadeh A . The car macrophage cells, a novel generation of chimeric antigen-based approach against solid tumors. biomark Res. (2023) 11:103. doi: 10.1186/s40364-023-00537-x, PMID: 38017494 PMC10685521

[26] Su S Lei A Wang X Lu H Wang S Yang Y . Induced car-macrophages as a novel therapeutic cell type for cancer immune cell therapies. Cells. (2022) 11:1652. doi: 10.3390/cells11101652, PMID: 35626689 PMC9139529

[27] Zhang WF Shao HW Wu FL Xie X Li ZM Bo HB . Influence of cell physiological state on gene delivery to T lymphocytes by chimeric adenovirus Ad5f35. Sci Rep. (2016) 6:22688. doi: 10.1038/srep22688, PMID: 26972139 PMC4789598

[28] Chen Y Zhu X Liu H Wang C Chen Y Wang H . The application of Her2 and Cd47 car-macrophage in ovarian cancer. J Trans Med. (2023) 21:654. doi: 10.1186/s12967-023-04479-8, PMID: 37740183 PMC10517545

[29] Zhang W Liu L Su H Liu Q Shen J Dai H . Chimeric antigen receptor macrophage therapy for breast tumours mediated by targeting the tumour extracellular matrix. Br J Cancer. (2019) 121:837–45. doi: 10.1038/s41416-019-0578-3, PMID: 31570753 PMC6889154

[30] Lei A Yu H Lu S Lu H Ding X Tan T . A second-generation M1-polarized car macrophage with antitumor efficacy. Nat Immunol. (2024) 25:102–16. doi: 10.1038/s41590-023-01687-8, PMID: 38012418

[31] Zhang L Tian L Dai X Yu H Wang J Lei A . Pluripotent stem cell-derived car-macrophage cells with antigen-dependent anti-cancer cell functions. J Hematol Oncol. (2020) 13:153. doi: 10.1186/s13045-020-00983-2, PMID: 33176869 PMC7656711

[32] Kang M Lee SH Kwon M Byun J Kim D Kim C . Nanocomplex-mediated *in vivo* programming to chimeric antigen receptor-M1 macrophages for cancer therapy. Advanced materials (Deerfield Beach Fla). (2021) 33:e2103258. doi: 10.1002/adma.202103258, PMID: 34510559

[33] Zhou J-e Zhou Z Wang Z Sun L Li F Tang Y . Lipid nanoparticles produce chimeric antigen receptor macrophages (Car-M) in situ for the treatment of solid tumors. Nano Today. (2025) 61:102610. doi: 10.1016/j.nantod.2024.102610

[34] Duan Z Li Z Wang Z Chen C Luo Y . Chimeric antigen receptor macrophages activated through Tlr4 or Ifn-Γ Receptors suppress breast cancer growth by targeting Vegfr2. Cancer immunology immunotherapy: CII. (2023) 72:3243–57. doi: 10.1007/s00262-023-03490-8, PMID: 37438548 PMC10992605

[35] Zhang Y Hu R Xie X Li Y . Expanding the frontier of car therapy: comparative insights into Car-T, Car-Nk, Car-M, and Car-Dc approaches. Ann Hematol. (2025) 104:4305–4317. doi: 10.1007/s00277-025-06538-0, PMID: 40928667 PMC12552347

[36] Mackensen A Müller F Mougiakakos D Böltz S Wilhelm A Aigner M . Anti-Cd19 Car T cell therapy for refractory systemic lupus erythematosus. Nat Med. (2022) 28:2124–32. doi: 10.1038/s41591-022-02017-5, PMID: 36109639

[37] Alabanza L Pegues M Geldres C Shi V Wiltzius JJW Sievers SA . Function of novel anti-Cd19 chimeric antigen receptors with human variable regions is affected by hinge and transmembrane domains. Mol Ther. (2017) 25:2452–65. doi: 10.1016/j.ymthe.2017.07.013, PMID: 28807568 PMC5675490

[38] Dingfelder J Aigner M Taubmann J Minopoulou I Park S Kaplan CD . Fully human anti-Cd19 Car T cells derived from systemic lupus erythematosus patients exhibit cytotoxicity with reduced inflammatory cytokine production. Transplant Cell Ther. (2024) 30:582. doi: 10.1016/j.jtct.2024.03.023, PMID: 38548226

[39] Sumitomo S Nagafuchi Y Tsuchida Y Tsuchiya H Ota M Ishigaki K . Transcriptome analysis of peripheral blood from patients with rheumatoid arthritis: A systematic review. Inflammation regeneration. (2018) 38:21. doi: 10.1186/s41232-018-0078-5, PMID: 30410636 PMC6217768

[40] Myers LK Ouyang YX Patel JR Odens HH Woo-Rasberry V Park J . Role of citrullinated collagen in autoimmune arthritis. Int J Mol Sci. (2022) 23:9833. doi: 10.3390/ijms23179833, PMID: 36077232 PMC9456437

[41] Whittington KB Prislovsky A Beaty J Albritton L Radic M Rosloniec EF . Cd8(+) T cells expressing an Hla-Dr1 chimeric antigen receptor target autoimmune Cd4(+) T cells in an antigen-specific manner and inhibit the development of autoimmune arthritis. J Immunol (Baltimore Md: 1950). (2022) 208:16–26. doi: 10.4049/jimmunol.2100643, PMID: 34819392 PMC8702470

[42] Wang H . A review of the effects of collagen treatment in clinical studies. Polymers. (2021) 13:3868. doi: 10.3390/polym13223868, PMID: 34833168 PMC8620403

[43] He Y Aoun M Xu Z Holmdahl R . Shift in perspective: autoimmunity protecting against rheumatoid arthritis. Ann rheumatic Dis. (2024) 83:550–5. doi: 10.1136/ard-2023-225237, PMID: 38413169

[44] Zhang B Wang Y Yuan Y Sun J Liu L Huang D . *In vitro* elimination of autoreactive B cells from rheumatoid arthritis patients by universal chimeric antigen receptor T cells. Ann rheumatic Dis. (2021) 80:176–84. doi: 10.1136/annrheumdis-2020-217844, PMID: 32998865

[45] Liu X Zhao J Shi C Liu Z Shen H Dang J . Construction of cii-specific Car-T to explore the cytokine cascades between cartilage-reactive T cells and chondrocytes. Front Immunol. (2020) 11:568741. doi: 10.3389/fimmu.2020.568741, PMID: 33343563 PMC7746615

[46] Alivernini S MacDonald L Elmesmari A Finlay S Tolusso B Gigante MR . Distinct synovial tissue macrophage subsets regulate inflammation and remission in rheumatoid arthritis. Nat Med. (2020) 26:1295–306. doi: 10.1038/s41591-020-0939-8, PMID: 32601335

[47] Yang Y Guo L Wang Z Liu P Liu X Ding J . Targeted silver nanoparticles for rheumatoid arthritis therapy via macrophage apoptosis and re-polarization. Biomaterials. (2021) 264:120390. doi: 10.1016/j.biomaterials.2020.120390, PMID: 32980634

[48] Cutolo M Campitiello R Gotelli E Soldano S . The role of M1/M2 macrophage polarization in rheumatoid arthritis synovitis. Front Immunol. (2022) 13:867260. doi: 10.3389/fimmu.2022.867260, PMID: 35663975 PMC9161083

[49] Jin X Xu Q Pu C Zhu K Lu C Jiang Y . Therapeutic efficacy of anti-Cd19 Car-T cells in a mouse model of systemic lupus erythematosus. Cell Mol Immunol. (2021) 18:1896–903. doi: 10.1038/s41423-020-0472-1, PMID: 32472023 PMC8322088

[50] Wilson JJ Wei J Daamen AR Sears JD Bechtel E Mayberry CL . Glucose oxidation-dependent survival of activated B cells provides a putative novel therapeutic target for lupus treatment. iScience. (2023) 26:107487. doi: 10.1016/j.isci.2023.107487, PMID: 37636066 PMC10448027

[51] Wang D Wang X Tan B Wen X Ye S Wu Y . Allogeneic Cd19-targeted Car-T therapy in refractory systemic lupus erythematosus achieved durable remission. Med (New York NY). (2025) 6:100749. doi: 10.1016/j.medj.2025.100749, PMID: 40446794

[52] Kretschmann S Völkl S Reimann H Krönke G Schett G Achenbach S . Successful generation of Cd19 chimeric antigen receptor T cells from patients with advanced systemic lupus erythematosus. Transplant Cell Ther. (2023) 29:27–33. doi: 10.1016/j.jtct.2022.10.004, PMID: 36241147

[53] Cortés JR Barba P Linares M An Open-label, Multi-center, Phase 1/2 Study to Assess Safety, Efficacy and Cellular Kinetics of YTB323, a Rapid Manufacturing CAR-T Cell Therapy Targeting CD19 on B Cells, for Severe Refractory Systemic Lupus Erythematosus: Preliminary Results. Arthritis Rheumatola. (2023) San Diego, CA: American College of Rheumatology (ACR) Convergence (2023) 75.

[54] Wang Z Zhao M Yin J Liu L Hu L Huang Y . E4bp4-mediated inhibition of T follicular helper cell differentiation is compromised in autoimmune diseases. J Clin Invest. (2020) 130:3717–33. doi: 10.1172/jci129018, PMID: 32191636 PMC7324209

[55] Dong X Antao OQ Song W Sanchez GM Zembrzuski K Koumpouras F . Type I interferon-activated Stat4 regulation of follicular helper T cell-dependent cytokine and immunoglobulin production in lupus. Arthritis Rheumatol (Hoboken NJ). (2021) 73:478–89. doi: 10.1002/art.41532, PMID: 33512094 PMC7914134

[56] Zhang X Lindwall E Gauthier C Lyman J Spencer N Alarakhia A . Circulating Cxcr5+Cd4+Helper T cells in systemic lupus erythematosus patients share phenotypic properties with germinal center follicular helper T cells and promote antibody production. Lupus. (2015) 24:909–17. doi: 10.1177/0961203314567750, PMID: 25654980

[57] Manfrè V Cafaro G Riccucci I Zabotti A Perricone C Bootsma H . One year in review 2020: comorbidities, diagnosis and treatment of primary Sjögren’s syndrome. Clin Exp Rheumatol. (2020) 38 Suppl 126:10–22., PMID: 32940212

[58] Ramos-Casals M Brito-Zerón P Kostov B Sisó-Almirall A Bosch X Buss D . Google-driven search for big data in autoimmune geoepidemiology: analysis of 394,827 patients with systemic autoimmune diseases. Autoimmun Rev. (2015) 14:670–9. doi: 10.1016/j.autrev.2015.03.008, PMID: 25842074

[59] Sandhya P Kurien BT Danda D Scofield RH . Update on pathogenesis of Sjogren’s syndrome. Curr Rheumatol Rev. (2017) 13:5–22. doi: 10.2174/1573397112666160714164149, PMID: 27412602 PMC5280579

[60] Srivastava A Makarenkova HP . Innate immunity and biological therapies for the treatment of Sjögren’s syndrome. Int J Mol Sci. (2020) 21:9172. doi: 10.3390/ijms21239172, PMID: 33271951 PMC7730146

[61] Verschuuren JJ Palace J Murai H Tannemaat MR Kaminski HJ Bril V . Advances and ongoing research in the treatment of autoimmune neuromuscular junction disorders. Lancet Neurol. (2022) 21:189–202. doi: 10.1016/s1474-4422(21)00463-4, PMID: 35065041

[62] Zhang Y Liu D Zhang Z Huang X Cao J Wang G . Bispecific Bcma/Cd19 targeted Car-T cell therapy forces sustained disappearance of symptoms and anti-acetylcholine receptor antibodies in refractory myasthenia gravis: A case report. J Neurol. (2024) 271:4655–9. doi: 10.1007/s00415-024-12367-4, PMID: 38602546

[63] Motte J Sgodzai M Schneider-Gold C Steckel N Mika T Hegelmaier T . Treatment of concomitant myasthenia gravis and Lambert-Eaton myasthenic syndrome with autologous Cd19-targeted Car T cells. Neuron. (2024) 112:1757–63.e2. doi: 10.1016/j.neuron.2024.04.014, PMID: 38697115

[64] Granit V Benatar M Kurtoglu M Miljković MD Chahin N Sahagian G . Safety and clinical activity of autologous Rna chimeric antigen receptor T-cell therapy in myasthenia gravis (Mg-001): A prospective, multicentre, open-label, non-randomised phase 1b/2a study. Lancet Neurol. (2023) 22:578–90. doi: 10.1016/s1474-4422(23)00194-1, PMID: 37353278 PMC10416207

[65] Tian DS Qin C Dong MH Heming M Zhou LQ Wang W . B cell lineage reconstitution underlies Car-T cell therapeutic efficacy in patients with refractory myasthenia gravis. EMBO Mol Med. (2024) 16:966–87. doi: 10.1038/s44321-024-00043-z, PMID: 38409527 PMC11018773

[66] Qin C Dong MH Zhou LQ Wang W Cai SB You YF . Single-cell analysis of refractory anti-Srp necrotizing myopathy treated with anti-Bcma Car-T cell therapy. Proc Natl Acad Sci United States America. (2024) 121:e2315990121. doi: 10.1073/pnas.2315990121, PMID: 38289960 PMC10861907

[67] Pecher AC Hensen L Klein R Schairer R Lutz K Atar D . Cd19-targeting Car T cells for myositis and interstitial lung disease associated with antisynthetase syndrome. JAMA. (2023) 329:2154–62. doi: 10.1001/jama.2023.8753, PMID: 37367976 PMC10300719

[68] Chu F Shi M Zheng C Shen D Zhu J Zheng X . The roles of macrophages and microglia in multiple sclerosis and experimental autoimmune encephalomyelitis. J neuroimmunology. (2018) 318:1–7. doi: 10.1016/j.jneuroim.2018.02.015, PMID: 29606295

[69] Yi J Miller AT Archambault AS Jones AJ Bradstreet TR Bandla S . Antigen-specific depletion of Cd4(+) T cells by Car T cells reveals distinct roles of higher- and lower-affinity Tcrs during autoimmunity. Sci Immunol. (2022) 7:eabo0777. doi: 10.1126/sciimmunol.abo0777, PMID: 36206355 PMC9867937

[70] Frikeche J David M Mouska X Treguer D Cui Y Rouquier S . Mog-specific Car Tregs: A novel approach to treat multiple sclerosis. J Neuroinflamm. (2024) 21:268. doi: 10.1186/s12974-024-03262-w, PMID: 39428507 PMC11490997

[71] Gupta S Simic M Sagan SA Shepherd C Duecker J Sobel RA . Car-T cell-mediated B-cell depletion in central nervous system autoimmunity. Neurology(R) neuroimmunology Neuroinflamm. (2023) 10:1–10. doi: 10.1212/nxi.0000000000200080, PMID: 36657993 PMC9853314

[72] Moorman CD Yu S Briseno CG Phee H Sahoo A Ramrakhiani A . Car-T cells and Car-Tregs targeting conventional type-1 dendritic cell suppress experimental autoimmune encephalomyelitis. Front Immunol. (2023) 14:1235222. doi: 10.3389/fimmu.2023.1235222, PMID: 37965348 PMC10641730

[73] Qin C Zhang M Mou DP Zhou LQ Dong MH Huang L . Single-cell analysis of anti-Bcma Car T cell therapy in patients with central nervous system autoimmunity. Sci Immunol. (2024) 9:eadj9730. doi: 10.1126/sciimmunol.adj9730, PMID: 38728414

[74] Qin C Tian DS Zhou LQ Shang K Huang L Dong MH . Anti-Bcma Car T-cell therapy Ct103a in relapsed or refractory Aqp4-Igg seropositive neuromyelitis optica spectrum disorders: phase 1 trial interim results. Signal transduction targeted Ther. (2023) 8:5. doi: 10.1038/s41392-022-01278-3, PMID: 36596762 PMC9810610

[75] Yang K Zhang Y Ding J Li Z Zhang H Zou F . Autoimmune Cd8+ T cells in type 1 diabetes: from single-cell Rna sequencing to T-cell receptor redirection. Front Endocrinol. (2024) 15:1377322. doi: 10.3389/fendo.2024.1377322, PMID: 38800484 PMC11116783

[76] Zhang L Sosinowski T Cox AR Cepeda JR Sekhar NS Hartig SM . Chimeric antigen receptor (Car) T cells targeting a pathogenic Mhc class ii: peptide complex modulate the progression of autoimmune diabetes. J Autoimmun. (2019) 96:50–8. doi: 10.1016/j.jaut.2018.08.004, PMID: 30122420 PMC6541442

[77] Radichev IA Yoon J Scott DW Griffin K Savinov AY . Towards antigen-specific Tregs for type 1 diabetes: construction and functional assessment of pancreatic endocrine marker, Hpi2-based chimeric antigen receptor. Cell Immunol. (2020) 358:104224. doi: 10.1016/j.cellimm.2020.104224, PMID: 33068914 PMC7655659

[78] Bluestone JA Buckner JH Fitch M Gitelman SE Gupta S Hellerstein MK . Type 1 diabetes immunotherapy using polyclonal regulatory T cells. Sci Trans Med. (2015) 7:315ra189. doi: 10.1126/scitranslmed.aad4134, PMID: 26606968 PMC4729454

[79] Huang Q Zhu J . Regulatory T cell-based therapy in type 1 diabetes: latest breakthroughs and evidence. Int Immunopharmacol. (2024) 140:112724. doi: 10.1016/j.intimp.2024.112724, PMID: 39098233

[80] Duan H Jiang Z Chen L Bai X Cai H Yang X . Tshr-based chimeric antigen receptor T cell specifically deplete auto-reactive B lymphocytes for treatment of autoimmune thyroid disease. Int Immunopharmacol. (2023) 124:110873. doi: 10.1016/j.intimp.2023.110873, PMID: 37690235

[81] Cheever A Lindsay HG Kang CC Hansen M Demars K O’Neill KL . Chimeric autoantibody receptor T cells specifically eliminate Graves’ Disease autoreactive B cells. Front Immunol. (2025) 16:1562662. doi: 10.3389/fimmu.2025.1562662, PMID: 40264771 PMC12011768

[82] Ratajczak K Grel H Olejnik P Jakiela S Stobiecka M . Current progress, strategy, and prospects of Pd-1/Pdl-1 immune checkpoint biosensing platforms for cancer diagnostics, therapy monitoring, and drug screening. (2023) 240:115644. doi: 10.1016/j.bios.2023.115644, PMID: 37660460

[83] Wei SC Duffy CR Allison JP . Fundamental mechanisms of immune checkpoint blockade therapy. Cancer Discov. (2018) 8:1069–86. doi: 10.1158/2159-8290.Cd-18-0367, PMID: 30115704

[84] McGowan E Lin Q Ma G Yin H Chen S Lin YJB . Pd-1 disrupted Car-T cells in the treatment of solid tumors: promises and challenges. Biomed Pharmacother. (2020) 121:109625. doi: 10.1016/j.biopha.2019.109625, PMID: 31733578

[85] Zhu HX Yang SH Gao CY Bian ZH Chen XM Huang RR . Targeting pathogenic Cd8(+) tissue-resident T cells with chimeric antigen receptor therapy in murine autoimmune cholangitis. Nat Commun. (2024) 15:2936. doi: 10.1038/s41467-024-46654-5, PMID: 38580644 PMC10997620

[86] Han F Jiang Z Guo Q Li Y Li C Liang X . Cd19 chimeric antigen receptor-T cell therapy in murine immune thrombocytopenia. Br J haematology. (2025) 206:1430–1442. doi: 10.1111/bjh.20061, PMID: 40139759

[87] Wang J Du H Xie W Bi J Zhang H Liu X . Car-macrophage therapy alleviates myocardial ischemia-reperfusion injury. Circ Res. (2024) 135:1161–74. doi: 10.1161/circresaha.124.325212, PMID: 39465245

[88] Dai H Zhu C Huai Q Xu W Zhu J Zhang X . Chimeric antigen receptor-modified macrophages ameliorate liver fibrosis in preclinical models. J Hepatol. (2024) 80:913–27. doi: 10.1016/j.jhep.2024.01.034, PMID: 38340812

[89] Mao Y Yao C Zhang S Zeng Q Wang J Sheng C . Targeting fibroblast activation protein with chimeric antigen receptor macrophages. Biochem Pharmacol. (2024) 230:116604. doi: 10.1016/j.bcp.2024.116604, PMID: 39489223

[90] Wang W Hu K Xue J Chen J Du X Zhao T . *In vivo* Fap-Car macrophages enhance chemotherapy and immunotherapy against pancreatic cancer by removing the fibrosis barrier. J Controlled release: Off J Controlled Release Soc. (2025) 384:113888. doi: 10.1016/j.jconrel.2025.113888, PMID: 40425095

[91] Knezevic NN Candido KD Vlaeyen JWS Van Zundert J Cohen SP . Low back pain. Lancet (London England). (2021) 398:78–92. doi: 10.1016/s0140-6736(21)00733-9, PMID: 34115979

[92] Zhou X Zhu D Wu D Li G Liang H Zhang W . Microneedle delivery of Car-M-like engineered macrophages alleviates intervertebral disc degeneration through enhanced efferocytosis capacity. Cell Rep Med. (2025) 6:102079. doi: 10.1016/j.xcrm.2025.102079, PMID: 40199328 PMC12047514

[93] Li YR Lyu Z Chen Y Fang Y Yang L . Frontiers in Car-T cell therapy for autoimmune diseases. Trends Pharmacol Sci. (2024) 45:839–57. doi: 10.1016/j.tips.2024.07.005, PMID: 39147651

[94] Müller F Taubmann J Bucci L Wilhelm A Bergmann C Völkl S . Cd19 Car T-cell therapy in autoimmune disease — a case series with follow-up. N Engl J Med. (2024) 390:687–700. doi: 10.1056/NEJMoa2308917, PMID: 38381673

[95] Kattamuri L Mohan Lal B Vojjala N Jain M Sharma K Jain S . Safety and efficacy of Car-T cell therapy in patients with autoimmune diseases: A systematic review. Rheumatol Int. (2025) 45:18. doi: 10.1007/s00296-024-05772-5, PMID: 39754644

[96] Zhang Z Xu Q Huang L . B cell depletion therapies in autoimmune diseases: monoclonal antibodies or chimeric antigen receptor-based therapy? Front Immunol. (2023) 14:1126421. doi: 10.3389/fimmu.2023.1126421, PMID: 36855629 PMC9968396

[97] Crickx E Weill JC Reynaud CA Mahévas M . Anti-Cd20-mediated B-cell depletion in autoimmune diseases: successes, failures and future perspectives. Kidney Int. (2020) 97:885–93. doi: 10.1016/j.kint.2019.12.025, PMID: 32229095

[98] Merrill JT Guthridge J Smith M June J Koumpouras F Machua W . Obexelimab in systemic lupus erythematosus with exploration of response based on gene pathway co-expression patterns: A double-blind, randomized, placebo-controlled, phase 2 trial. Arthritis Rheumatol (Hoboken NJ). (2023) 75:2185–94. doi: 10.1002/art.42652, PMID: 37459248

[99] Bodansky A Yu DJ Rallistan A Kalaycioglu M Boonyaratanakornkit J Green DJ . Unveiling the proteome-wide autoreactome enables enhanced evaluation of emerging Car T cell therapies in autoimmunity. J Clin Invest. (2024) 134:e180012. doi: 10.1172/jci180012, PMID: 38753445 PMC11213466

[100] Wang K Wei G Liu D . Cd19: A biomarker for B cell development, lymphoma diagnosis and therapy. Exp Hematol Oncol. (2012) 1:36. doi: 10.1186/2162-3619-1-36, PMID: 23210908 PMC3520838

[101] O’Connor BP Raman VS Erickson LD Cook WJ Weaver LK Ahonen C . Bcma is essential for the survival of long-lived bone marrow plasma cells. J Exp Med. (2004) 199:91–8. doi: 10.1084/jem.20031330, PMID: 14707116 PMC1887725

[102] Müller F Wirsching A Hagen M Völkl S Tur C Raimondo MG . Bcma-Car T-cells in a patient with relapsing idiopathic inflammatory myositis after Cd19-Car T-cells. Nat Med. (2025) 31:1793–1797. doi: 10.1038/s41591-025-03718-3, PMID: 40245922 PMC12176613

[103] Liu Q Li J Zheng H Yang S Hua Y Huang N . Adoptive cellular immunotherapy for solid neoplasms beyond Car-T. Mol Cancer. (2023) 22:28. doi: 10.1186/s12943-023-01735-9, PMID: 36750830 PMC9903509

[104] Liu M Liu J Liang Z Dai K Gan J Wang Q . Car-macrophages and Car-T cells synergistically kill tumor cells *in vitro*. Cells. (2022) 11:3692. doi: 10.3390/cells11223692, PMID: 36429120 PMC9688246

[105] Mondal N Silva M Castano AP Maus MV Sackstein R . Glycoengineering of chimeric antigen receptor (Car) T-cells to enforce E-selectin binding. J Biol Chem. (2019) 294:18465–74. doi: 10.1074/jbc.RA119.011134, PMID: 31628196 PMC6885642

[106] Li X Wang X Wang H Zuo D Xu J Feng Y . A clinical study of autologous chimeric antigen receptor macrophage targeting mesothelin shows safety in ovarian cancer therapy. J Hematol Oncol. (2024) 17:116. doi: 10.1186/s13045-024-01635-5, PMID: 39609867 PMC11603993

[107] Zhu X Fang Y Chen Y Chen Y Hong W Wei W . Interaction of tumor-associated microglia/macrophages and cancer stem cells in glioma. Life Sci. (2023) 320:121558. doi: 10.1016/j.lfs.2023.121558, PMID: 36889666

[108] Wang S Yang Y Ma P Zha Y Zhang J Lei A . Car-macrophage: an extensive immune enhancer to fight cancer. EBioMedicine. (2022) 76:103873. doi: 10.1016/j.ebiom.2022.103873, PMID: 35152151 PMC8844597

[109] Fiorenza S Ritchie DS Ramsey SD Turtle CJ Roth JA . Value and affordability of Car T-cell therapy in the United States. Bone marrow Transplant. (2020) 55:1706–15. doi: 10.1038/s41409-020-0956-8, PMID: 32474570

[110] Lee SM Kang CH Choi SU Kim Y Hwang JY Jeong HG . A chemical switch system to modulate chimeric antigen receptor T cell activity through proteolysis-targeting chimaera technology. ACS synthetic Biol. (2020) 9:987–92. doi: 10.1021/acssynbio.9b00476, PMID: 32352759

[111] Sun N Wang C Edwards W Wang Y Lu XL Gu C . Nanoneedle-based electroporation for efficient manufacturing of human primary chimeric antigen receptor regulatory T-cells. Advanced Sci (Weinheim Baden-Wurttemberg Germany). (2025) 12:1–13. doi: 10.1002/advs.202416066, PMID: 40231643 PMC12140303

[112] Brezinger-Dayan K Itzhaki O Melnichenko J Kubi A Zeltzer LA Jacoby E . Impact of cryopreservation on Car T production and clinical response. Front Oncol. (2022) 12:1024362. doi: 10.3389/fonc.2022.1024362, PMID: 36276077 PMC9582437

[113] Milone MC O’Doherty U . Clinical use of lentiviral vectors. Leukemia. (2018) 32:1529–41. doi: 10.1038/s41375-018-0106-0, PMID: 29654266 PMC6035154

[114] Xiao Y Zhu T Chen Z Huang X . Lung metastasis and recurrence is mitigated by car macrophages, in-situ-generated from Mrna delivered by small extracellular vesicles. Nat Commun. (2025) 16:7166. doi: 10.1038/s41467-025-62506-2, PMID: 40759657 PMC12322094

[115] Chuang ST Stein JB Nevins S Kilic Bektas C Choi HK Ko WK . Enhancing car macrophage efferocytosis via surface engineered lipid nanoparticles targeting Lxr signaling. Advanced materials (Deerfield Beach Fla). (2024) 36:e2308377. doi: 10.1002/adma.202308377, PMID: 38353580 PMC11081841

[116] Zhang H Huo Y Zheng W Li P Li H Zhang L . Silencing of Sirpα Enhances the antitumor efficacy of Car-M in solid tumors. Cell Mol Immunol. (2024) 21:1335–49. doi: 10.1038/s41423-024-01220-3, PMID: 39379603 PMC11527885

[117] Cao Q Wang Y Chen J Wang R Chen T Gloss B . Targeting inflammation with chimeric antigen receptor macrophages using a signal switch. Nat Biomed Eng. (2025) 9:1502–1516. doi: 10.1038/s41551-025-01387-8, PMID: 40335685 PMC12443588

[118] Ghassemi S Nunez-Cruz S O’Connor RS Fraietta JA Patel PR Scholler J . Reducing ex vivo culture improves the antileukemic activity of chimeric antigen receptor (Car) T cells. Cancer Immunol Res. (2018) 6:1100–9. doi: 10.1158/2326-6066.Cir-17-0405, PMID: 30030295 PMC8274631

[119] Mougiakakos D Sengupta R Gold R Schroers R Haghikia A Lorente M . Successful generation of fully human, second generation, anti-Cd19 Car T cells for clinical use in patients with diverse autoimmune disorders. Cytotherapy. (2025) 27:236–46. doi: 10.1016/j.jcyt.2024.09.008, PMID: 39530971

[120] Del Bufalo F Becilli M Rosignoli C De Angelis B Algeri M Hanssens L . Allogeneic, donor-derived, second-generation, Cd19-directed Car-T cells for the treatment of pediatric relapsed/refractory Bcp-all. Blood. (2023) 142:146–57. doi: 10.1182/blood.2023020023, PMID: 37172203

[121] Bader P . Allo or auto Car-T cells for refractory Bcp-all? Blood. (2023) 142:122–4. doi: 10.1182/blood.2023020814, PMID: 37440268

[122] Teachey DT Rheingold SR Maude SL Zugmaier G Barrett DM Seif AE . Cytokine release syndrome after blinatumomab treatment related to abnormal macrophage activation and ameliorated with cytokine-directed therapy. Blood. (2013) 121:5154–7. doi: 10.1182/blood-2013-02-485623, PMID: 23678006 PMC4123427

[123] Xiao X Huang S Chen S Wang Y Sun Q Xu X . Mechanisms of cytokine release syndrome and neurotoxicity of Car T-cell therapy and associated prevention and management strategies. J Exp Clin Cancer research: CR. (2021) 40:367. doi: 10.1186/s13046-021-02148-6, PMID: 34794490 PMC8600921

[124] Bijlsma JWJ Welsing PMJ Woodworth TG Middelink LM Pethö-Schramm A Bernasconi C . Early rheumatoid arthritis treated with tocilizumab, methotrexate, or their combination (U-act-early): A multicentre, randomised, double-blind, double-dummy, strategy trial. Lancet (London England). (2016) 388:343–55. doi: 10.1016/s0140-6736(16)30363-4, PMID: 27287832

[125] Le RQ Li L Yuan W Shord SS Nie L Habtemariam BA . Fda approval summary: tocilizumab for treatment of chimeric antigen receptor T cell-induced severe or life-threatening cytokine release syndrome. oncologist. (2018) 23:943–7. doi: 10.1634/theoncologist.2018-0028, PMID: 29622697 PMC6156173

[126] Strati P Ahmed S Furqan F Fayad LE Lee HJ Iyer SP . Prognostic impact of corticosteroids on efficacy of chimeric antigen receptor T-cell therapy in large B-cell lymphoma. Blood. (2021) 137:3272–6. doi: 10.1182/blood.2020008865, PMID: 33534891 PMC8351896

[127] Liu D Xu X Dai Y Zhao X Bao S Ma W . Blockade of aim2 inflammasome or A1-ar ameliorates Il-1β Release and macrophage-mediated immunosuppression induced by Car-T treatment. J immunotherapy Cancer. (2021) 9:1–14. doi: 10.1136/jitc-2020-001466, PMID: 33414262 PMC7797290

[128] Yoon SH Huh BK Abdi S Javed S . The efficacy of high-intensity laser therapy in wound healing: A narrative review. Lasers Med Sci. (2024) 39:208. doi: 10.1007/s10103-024-04146-4, PMID: 39096352

[129] Wang X Wu X Tan B Zhu L Zhang Y Lin L . Allogeneic Cd19-targeted Car-T therapy in patients with severe myositis and systemic sclerosis. Cell. (2024) 187:4890–904.e9. doi: 10.1016/j.cell.2024.06.027, PMID: 39013470

[130] Hu Y Zhou Y Zhang M Ge W Li Y Yang L . Crispr/Cas9-engineered universal Cd19/Cd22 dual-targeted Car-T cell therapy for relapsed/refractory B-cell acute lymphoblastic leukemia. Clin Cancer Res. (2021) 27:2764–72. doi: 10.1158/1078-0432.Ccr-20-3863, PMID: 33627493

[131] Shirzadian M Moori S Rabbani R Rahbarizadeh F . Synnotch Car-T cell, when synthetic biology and immunology meet again. Front Immunol. (2025) 16:1545270. doi: 10.3389/fimmu.2025.1545270, PMID: 40308611 PMC12040928

[132] Chen Z Shu J Hu Y Mei H . Synergistic integration of Mrna-Lnp with car-engineered immune cells: pioneering progress in immunotherapy. Mol Ther. (2024) 32:3772–92. doi: 10.1016/j.ymthe.2024.09.019, PMID: 39295145 PMC11573621

[133] Lyu Z Niu S Fang Y Chen Y Li YR Yang L . Addressing graft-versus-host disease in allogeneic cell-based immunotherapy for cancer. Exp Hematol Oncol. (2025) 14:66. doi: 10.1186/s40164-025-00654-3, PMID: 40317083 PMC12046680

[134] Sena LA Li S Jairaman A Prakriya M Ezponda T Hildeman DA . Mitochondria are required for antigen-specific T cell activation through reactive oxygen species signaling. Immunity. (2013) 38:225–36. doi: 10.1016/j.immuni.2012.10.020, PMID: 23415911 PMC3582741

[135] Vardhana SA Hwee MA Berisa M Wells DK Yost KE King B . Impaired mitochondrial oxidative phosphorylation limits the self-renewal of T cells exposed to persistent antigen. Nat Immunol. (2020) 21:1022–33. doi: 10.1038/s41590-020-0725-2, PMID: 32661364 PMC7442749

[136] Laniewski NG Grayson JM . Antioxidant treatment reduces expansion and contraction of antigen-specific Cd8+ T cells during primary but not secondary viral infection. J Virol. (2004) 78:11246–57. doi: 10.1128/jvi.78.20.11246-11257.2004, PMID: 15452243 PMC521823

[137] Fisicaro P Barili V Montanini B Acerbi G Ferracin M Guerrieri F . Targeting mitochondrial dysfunction can restore antiviral activity of exhausted Hbv-specific Cd8 T cells in chronic hepatitis B. Nat Med. (2017) 23:327–36. doi: 10.1038/nm.4275, PMID: 28165481

[138] Zhang M Kong J Yin F Shi J Li J Qiu Z . Optimizing Car-T cell culture: differential effects of Il-2, Il-12, and Il-21 on Car-T cells. Cytokine. (2024) 184:156758. doi: 10.1016/j.cyto.2024.156758, PMID: 39299100

[139] Maalej KM Merhi M Inchakalody VP Mestiri S Alam M Maccalli C . Car-cell therapy in the era of solid tumor treatment: current challenges and emerging therapeutic advances. Mol Cancer. (2023) 22:20. doi: 10.1186/s12943-023-01723-z, PMID: 36717905 PMC9885707

[140] Patel AA Ginhoux F Yona S . Monocytes, macrophages, dendritic cells and neutrophils: an update on lifespan kinetics in health and disease. Immunology. (2021) 163:250–61. doi: 10.1111/imm.13320, PMID: 33555612 PMC8207393

[141] Li J Chen P Ma W . The next frontier in immunotherapy: potential and challenges of car-macrophages. Exp Hematol Oncol. (2024) 13:76. doi: 10.1186/s40164-024-00549-9, PMID: 39103972 PMC11302330

[142] Raziyeva K Kim Y Zharkinbekov Z Kassymbek K Jimi S Saparov A . Immunology of acute and chronic wound healing. Biomolecules. (2021) 11:700. doi: 10.3390/biom11050700, PMID: 34066746 PMC8150999

[143] Frykberg RG Banks J . Challenges in the treatment of chronic wounds. Adv Wound Care. (2015) 4:560–82. doi: 10.1089/wound.2015.0635, PMID: 26339534 PMC4528992

[144] Nazari M Taremi S Elahi R Mostanadi P Esmeilzadeh A . Therapeutic properties of M2 macrophages in chronic wounds: an innovative area of biomaterial-assisted M2 macrophage targeted therapy. Stem Cell Rev Rep. (2025) 21:390–422. doi: 10.1007/s12015-024-10806-3, PMID: 39556244

[145] Zhang H Wang Y Feng K Niu Q Xin Y Xuan S . Mir-146a-5p-enriched exosomes inhibit M1 macrophage activation and inflammatory response by targeting Cd80. Mol Biol Rep. (2024) 51:1133. doi: 10.1007/s11033-024-10088-5, PMID: 39514136

[146] Han YH Kim HJ Na H Nam MW Kim JY Kim JS . Rorα Induces Klf4-mediated M2 polarization in the liver macrophages that protect against nonalcoholic steatohepatitis. Cell Rep. (2017) 20:124–35. doi: 10.1016/j.celrep.2017.06.017, PMID: 28683306

[147] Liu Y Hu P Zheng Z Zhong D Xie W Tang Z . Photoresponsive vaccine-like car-M system with high-efficiency central immune regulation for inflammation-related depression. Advanced materials (Deerfield Beach Fla). (2022) 34:e2108525. doi: 10.1002/adma.202108525, PMID: 34897839

[148] Peng Y Zhou M Yang H Qu R Qiu Y Hao J . Regulatory mechanism of M1/M2 macrophage polarization in the development of autoimmune diseases. Mediators Inflammation. (2023) 2023:8821610. doi: 10.1155/2023/8821610, PMID: 37332618 PMC10270764

[149] Myers KV Amend SR Pienta KJ . Targeting Tyro3, Axl and Mertk (Tam receptors): implications for macrophages in the tumor microenvironment. Mol Cancer. (2019) 18:94. doi: 10.1186/s12943-019-1022-2, PMID: 31088471 PMC6515593

